# Data describing child development at 6 years after maternal cancer diagnosis and treatment during pregnancy

**DOI:** 10.1016/j.dib.2020.106209

**Published:** 2020-08-21

**Authors:** Mathilde van Gerwen, Tineke Vandenbroucke, Magali Verheecke, Kristel Van Calsteren, Michael J. Halaska, Monica Fumagalli, Robert Fruscio, Amarendra Gandhi, Margreet Veening, Lieven Lagae, Petronella B. Ottevanger, Jens-Uwe Voigt, Jorine de Haan, Mina M. Gziri, Charlotte Maggen, Luc Mertens, Gunnar Naulaers, Laurence Claes, Frédéric Amant, International Network on Cancer, Infertility and Pregnancy (INCIP)

**Affiliations:** a Center for Gynecologic Oncology, Netherlands Cancer Institute/Antoni van Leeuwenhoek Hospital, Amsterdam, the Netherlands; b Princess Máxima Center for pediatric oncology, Utrecht, the Netherlands; c Department of Obstetrics and Gynecology, University Hospitals Leuven; d Department of Oncology, KU Leuven, Leuven, Belgium; e Department of Development and Regeneration, KU Leuven, Leuven, Belgium; f Department of Obstetrics and Gynecology, 3rd Medical Faculty Charles University Faculty Hospital Kralovske Vinohrady, Prague, Czech Republic; g Neonatal Intensive Care Unit, Fondazione IRCCS Ca’ Granda Ospedale Maggiore Policlinico and Department of Clinical Sciences and Community Health, University of Milan, Milan, Italy; h Clinic of Obstetrics and Gynecology, University of Milan-Bicocca and San Gerardo Hospital, Monza, Italy; i Department of Public Health and Primary Care, Faculty of Medicine, KU Leuven, Leuven and Data Scientist, Knowledge Center, SD Worx, Antwerp, Belgium; j Department of Pediatrics, University Hospitals Leuven, Leuven, Belgium; k Department of Internal Medicine, Radboud UMC Nijmegen, the Netherlands; l Department of Cardiology, University Hospitals Leuven, Leuven, Belgium; m Department of Obstetrics and Gynecology, Amsterdam UMC, Location VU University Medical Center, the Netherlands; n Department of Obstetrics, Cliniques Universitaires St Luc, Brussels, Belgium; o Department of Cardiology, The Hospital for Sick Children, University of Toronto, Toronto, ON, Canada; p Department of Neonatology, University Hospitals Leuven, Leuven, Belgium; q Faculty of Psychology and Educational Sciences, KU Leuven, Leuven, Belgium; r Department of Obstetrics and Gynecology, Amsterdam UMC, Location Amsterdam Medical Center and University of Amsterdam, the Netherlands

**Keywords:** Child development, Pregnancy, High-risk, Infant, Follow-up studies, Antineoplastic agents, Prenatal Exposure Delayed Effects

## Abstract

This manuscript is an accompanying resource of the original research article entitled “Child development at 6 years after maternal cancer diagnosis and treatment during pregnancy” and present data that compare the outcome of 6-year-old-children born to women diagnosed with cancer during pregnancy (with or without treatment during pregnancy) (study group) with children born after an uncomplicated pregnancy (control group). Oncological, obstetrical and neonatal data were collected. Neurodevelopment was examined by clinical evaluation and neuropsychological testing (including intelligence, attention and memory tests) and by general health and behavior questionnaires. Cardiac evaluation included electro- and echocardiography. Univariate analyses of covariance were used to investigate between-group differences. A subgroup analysis was performed in chemotherapy-exposed children versus controls and anthracycline-exposed versus controls. Additionally, the incidence of behaviour problems was compared to matched controls for children whose mothers died and for those with surviving mothers.

## Specifications Table

**Table:** 

Subject	Perinatology, Pediatrics and Child Health
Specific subject area	This manuscript represents a cohort of children prenatally exposed to maternal cancer, the associated stress, diagnostic imaging and treatments (including surgery, chemotherapy and radiotherapy).
Type of data	Tables Figures
How data were acquired	Study children underwent a clinical neurological and general paediatric examination and the parents of study and control children filled out a health questionnaire. Study and control children underwent a cardiac evaluation. Cardiac evaluation included a 12-lead electrocardiography (ECG) and a full echocardiographic evaluation looking for structural and functional parameters. Heart rate and rhythm, blood pressure, PR interval, QRS duration, QT duration, and QT corrected for the heart rate were measured by the same observer. A functional paediatric echocardiogram was performed according to the standards of the American Society of Echocardiography guidelines [1] on a Vivid E-9 scanner (GE Ultrasound, USA). Study and control children underwent a comprehensive neuropsychological assessment using the Wechsler Intelligence tests [2], [3], [4], [5], [6] or Snijders-Oomen Nonverbal Intelligence Test [7], Children's Memory Scale [8] and Amsterdam Neuropsychological Tasks [9]. The parents of study and control children filled out the Child Behaviour Checklist [10] to evaluate behavioural development.
Data format	Raw and analysed
Parameters for data collection	Study group: Between 2005 and 2018, women diagnosed with cancer during pregnancy and referred to one of the participating centers in Belgium (University Hospitals Leuven), The Netherlands (Amsterdam University Medical Center, University Medical Center Utrecht, Erasmus Medical Center Rotterdam, University Medical Center Groningen and Radboud University Medical Center Nijmegen), the Czech Republic (Faculty Hospital Motol, Charles University Prague) and Italy (Istituto Europeo di Oncologia Milan, Ospedale San Gerardo Monza) were prospectively (during pregnancy) or retrospectively (after delivery but before the child was 6 years old) invited to take part in the study. There were no exclusion criteria for study children. Parents signed the informed consent at the moment of inclusion. Control group: Control children were recruited in the participating countries.. Preterm born control children were recruited through the screening of birth lists from the participating hospitals. Children born full term were recruited by distributing information letters in schools and by advertising on the webpage of the hospital. All parents who were willing to let their child participate in the study first filled out a questionnaire on general health and prenatal history, in order to check if they met the inclusion criteria. Exclusion was based on all pregnancy-related (e.g. hypertension, severe preeclampsia, gestational diabetes with medical treatment, liver problems, epilepsy …) or neonatal problems (e.g. admission to a neonatal ward because of infections, long-term need of oxygen, malformations, brain lesions …) that may impact on child development. Immediate postnatal oxygen administration (CPAP) was not considered an exclusion criterion. Parents whose child met all the inclusion criteria signed the informed consent consecutively.
Description of data collection	All children were prospectively examined. Parents were contacted by e-mail and/or phone to invite their children at the predefined age of 6 years. Examinations were grouped together on one day at the participating center. Oncological, obstetrical and neonatal data were collected for each mother-child pair. Neuropsychological assessment, cardiac examinations and a clinical neurological and paediatric examination were performed. Parents filled out a questionnaire on behavioural development and on general health of their children. To optimize compliance, we maintained regular personal contact with the families (family meet and greet days, birthday cards for the children); expenses for transport and parking were reimbursed; and participants were given a €10 allowance per hour of investigation time (in Belgium) or a small present (in the other countries) to compensate for their time and effort.
Data source location	UZ Leuven, Department of Gynaecological Oncology Leuven, Belgium
Data accessibility	Raw data is hosted in Mendeley Data. DOI: 10.17632/2hzvcg8cd8.1 All additional information about the maternal cancer types and specific treatments, characteristics of the children, perinatal outcome and growth, cognitive development and behavior, cardiac evaluation and health problems is provided and summarized in supplementary material.
Related research article	Author's name: Tineke Vandenbroucke, Magali Verheecke, Mathilde van Gerwen, Kristel Van Calsteren, Michael J. Halaska, Monica Fumagalli, Robert Fruscio, M.D., Amarendra Gandhi, Margreet Veening, Lieven Lagae, Petronella B. Ottevanger, Jens-Uwe Voigt, Jorine de Haan, Mina M. Gziri, Charlotte Maggen, Luc Mertens, Gunnar Naulaers, Laurence Claes, Frédéric Amant Title: Child development at 6 years after maternal cancer diagnosis and treatment during pregnancy Journal: European Journal of Cancer https://doi.org/10.1016/j.ejca.2020.07.004

## Value of the Data

• To our knowledge, this study represents the largest cohort of children prenatally exposed to maternal cancer, the associated stress, diagnostic imaging and treatments (including surgery, chemotherapy and radiotherapy).
• The follow-up of 6 years after the event, the inclusion of a one-to-one matched comparison to non-exposed children and the extensive examination of health status, cognitive development and cardiac structure and functions add to a better understanding of the possible long-term sequelae for these children.
• The availability of long-term follow-up data may help patients and their families to make an informed decision about the continuation of pregnancy and the start of cancer treatment during pregnancy.
• As the outcomes were in general reassuring, our data show that in many cases, the risks of maternal cancer treatment during pregnancy do not outweigh the benefit of maternal treatment delay or the need for termination of pregnancy.

## 1 Data Description

Here we report on data about 6-year-old-children born to women diagnosed with cancer during pregnancy. The data include the clinical evaluation, scores for the neurocognitive tests and outcomes of the cardiac evaluation. Information about the maternal cancer types and specific treatments is provided in Tables 1 and 2. All additional information about the maternal cancer types and specific treatments, characteristics of the children, perinatal outcome and growth, cognitive development and behavior, cardiac evaluation and health problems is provided and summarized in supplementary material (eTable 1–27 and eFigure1–4).

**Table 1:** Maternal tumor types treated during pregnancy (127 mothers, 132 children).

Maternal malignancy	N mothers	% mothers	N mothers deceased	% mothers deceased
Breast cancer	69 (3 twin pregnancies)	54.3	12 (1 twin)	17.4
Hematological Malignancy	20	15.7	3	15.0
- Acute Lymphoid Leukemia	2	1.6	0	0.0
- Acute Myeloid Leukemia	5	3.9	0	0.0
- Chronic Myeloid Leukemia	2	1.6	2	100.0
- Hodgkin's Disease	5	3.9	0	0.0
- Non-Hodgkin's Disease	6	4.7	1	16.7
Cervical cancer	10 (1 twin pregnancy)	7.9	3	30.0
Ovarian cancer	10	7.9	1	10.0
Brain tumor	4	3.1	1	25.0
Oral cavity and oropharyngeal cavity cancer	4 (1 twin pregnancy)	3.1	0	0.0
Nasopharynx tumor	1	0.8	0	0.0
Gastric cancer	2	1.6	2	100.0
Colon cancer	1	0.8	1	100.0
Melanoma	2	1.6	0	0.0
Thyroid cancer	1	0.8	0	0.0
Soft tissue sarcoma	1	0.8	1	100.0
Kidney carcinoma	1	0.8	0	0.0
Lung cancer	1	0.8	1	100.0
TOTAL	127	100.0	25	19.7

**Table 2:** Chemotherapy regimens applied during pregnancy in 93 women (including 4 twin-pregnancies).

Chemotherapy scheme	N Cycles	Npatients	%patients
(F)AC†	75	22⁎⁎	23.7
(F)E(C)†	156	36⁎⁎	38.7
ABVD†	15	5	5.4
(R) - CHOP†	18	5	5.4
Cisplatin (± Epirubicin)†	45	9⁎⁎	9.7
Carboplatin (± 5-Fluorouracil) / Cisplatin (± 5-Fluorouracil)	6	3⁎⁎	3.2
Paclitaxel-Cis/Carboplatin	31	7	7.5
Paclitaxel/Docetaxel	27	12	12.9
Hovon 37 / 70 / 42A†	4	2	2.2
Temozolomide	4	1	1.1
Idarubicin-AraC†	5	2	2.2
5-Fluorouracil	3	1	1.1
CMF	1	1	1.1
TOTAL	390	106*	

Abbreviations: (F)AC, 5-fluorouracil, doxorubicin, cyclophosphamide; (F)E(C), 5-fluorouracil, epirubicin, cyclophosphamide; ABVD, doxorubicin, bleomycin, vinblastine, dacarbazine; (*R*)-CHOP, rituximab, cyclophosphamide, doxorubicin, vincristine, prednisolone; Hovon 37, cycle 1 prednisolone, vincristine, daunorubicin, L-aparginase, methotrexate, and cycle 2 cytarabine, mitoxantrone, intrathecal methotrexate; Hovon 70, cycle 1 prenisolone, vincristine, daunorubicin, cyclophosphamide, and cycle 2 methotrexate, 6-thioguanine, cytarabine, cyclophosphamide; Hovon 42A (only induction phase during pregnancy), amsacrine, cytarabine; AraC, cytarabine; CMF, cyclophosphamide, methotrexate, 5-fluorouracil;.

⁎ 13 patients received 2 different schemes; † including anthracyclines;.

⁎⁎ including 1 twin-pregnancy.

## 2 Experimental Design, Materials and Methods

### 2.1 Recruitment of study and control children

Between 2005 and 2018, women diagnosed with cancer during pregnancy and referred to one of the participating centers in Belgium (University Hospitals Leuven), The Netherlands (Amsterdam University Medical Center, University Medical Center Utrecht, Erasmus Medical Center Rotterdam, University Medical Center Groningen and Radboud University Medical Center Nijmegen), the Czech Republic (Faculty Hospital Motol, Charles University Prague) and Italy (Istituto Europeo di Oncologia Milan, Ospedale San Gerardo Monza) were prospectively (during pregnancy) or retrospectively (after delivery but before the child was 6 years old) invited to take part in the study. All children were prospectively examined. There were no exclusion criteria for study children. Parents signed the informed consent at the moment of inclusion. Parents were contacted by mail, e-mail and/or phone to invite their children at the time of follow-up. Denial of participation or drop-out were mainly due to the distance to the hospital, difficulties to reach the patient after moving out or death of the mother and fear of overload for the child due to the supplementary examinations. Participants were offered to do (part of) the neuropsychological assessment at home if the distance to the hospital was the main reason for drop-out. The examination of some children at home, together with the fact that some centers were not able to perform cardiologic examinations, contributes to the lower number of children included for the cardiac examinations. To optimize compliance, we maintain regular personal contact with families (family meet and greet days, birthday cards for the children); expenses for transport and parking were reimbursed; and participants were given a €10 allowance per hour of investigation time (in Belgium) or a small present (in the other countries) to compensate for their time and effort.

Control children for the cognitive and general health examinations and for the biometry results were recruited in Belgium, the Netherlands, the Czech Republic and Italy. Cardiac examinations were conducted prospectively in the same control children from Belgium as for the cognitive examinations. However, as some children were assessed at home for the neuropsychological tests if the distance to the hospital was a problem for the parents, we had to search for different controls for the cardiac examinations. Twenty-six controls were therefore recruited from an existing recently acquired database from Toronto to be able to match accurately for age and gender. The cardiologist responsible for the data analysis (L.M.) used to work in Belgium at the start of the study and recruited normal controls in Toronto after he moved there.

Preterm born children were recruited through the screening of birth lists from the participating hospitals. Children born full term were recruited by distributing information letters in schools and by advertising on the webpage of the hospital. All parents who were willing to let their child participate in the study first filled out a questionnaire on general health and prenatal history, in order to check if they met the inclusion criteria. Exclusion was based on all pregnancy-related (e.g. hypertension, severe preeclampsia, gestational diabetes with medical treatment, liver problems, epilepsy …) or neonatal problems (e.g. admission to a neonatal ward because of infections, long-term need of oxygen, malformations, brain lesions …) that may impact on child development. Immediate postnatal oxygen administration (CPAP) was not considered an exclusion criterion. Parents whose child met all the inclusion criteria signed the informed consent consecutively. Reasons for denial of participation or drop-out were the same as for the study children.

### 2.2 Description of the neuropsychological assessment protocol

The neuropsychological assessment consisted of two parts: an intelligence test of about 1 to 1,5 hour and several attention and memory tests of about 1 hour altogether.

Part 1: intelligence test

The following intelligence tests were used in our study:

Wechsler Preschool and Primary Scale of Intelligence – revised edition (WPPSI-R) [2] (*N* = 19)
Wechsler Preschool and Primary Scale of Intelligence – third edition (WPPSI-III) [3] (*N* = 195)
Wechsler Preschool and Primary Scale of Intelligence – fourth edition (WPPSI-IV) [4] (*N* = 2)
Wechsler Intelligence Scale for Children – third edition (WISC-III) [5] (*N* = 29)
Wechsler Intelligence Scale for Children – fourth edition (WISC-IV) [6] (*N* = 6)
Snijders-Oomen Nonverbal Intelligence Test (SON-R 2.5–7 years) [7] (*N* = 7)

Different intelligence tests were used due to several reasons:

1. Intelligence tests are regularly revised in order to provide test materials that are adapted to the daily life of today's children and in order to update the norms to correct for the Flynn effect (i.e., the increase of intelligence scores in many parts of the world over the 20th century). The long-term nature of our study therefore implies that tests are revised during the duration of the project.
2. The Wechsler intelligence tests are developed in the United States of America and further translated, adapted and validated in other countries and languages. This implies that certain editions or revisions are not (yet) available in all languages (Dutch, French, Italian, Czech) and countries. As our study is a multicenter international study, the currently used edition of the Wechsler test is not always the same in all participating countries.

For WPPSI-III and WISC-III, Full Scale Intelligence Quotient (FSIQ), Verbal IQ (VIQ), Performance IQ (PIQ) and Processing Speed (PS) were calculated. For WPPSI-R, only FSIQ, VIQ and PIQ were calculated, as this test does not provide a score for Processing Speed. For WPPSI-IV and WISC-IV, only FSIQ and PS were used, as these tests do not provide scores for VIQ and PIQ. For SON-R, the SON-IQ score was calculated and we used this value as the ‘Full Scale IQ’ score.

All IQ-tests used in this study have a mean score of 100 with a standard deviation of 15. Higher scores indicate more advanced development. Scores between 90 and 110 are considered average.

Correlations between Wechsler intelligence tests and editions are high. For example, *r* = 0.80 for Full Scale Intelligence (FSIQ) measured by means of the Dutch edition of WPPSI-III and WISC-III. [11] Correlations for Verbal Intelligence (VIQ), Performance Intelligence (PIQ) and Processing Speed are also high between the two tests (VIQ: *r* = 0.81, PIQ: *r* = 0.70, PS: *r* = 0.73).

Part 2: attention and memory tests

Four subtasks from the Amsterdam Neuropsychological Tasks (ANT) were used to evaluate different aspects of attention. [9] ANT is a computerized program which enables to measure not only the accuracy of responses but also the reaction times. Prior to each task, the subjects were given verbal instructions and were shown the stimulus material. Next, they received a practice trial before the test of each task.

3. ‘Baseline Speed’

This task assesses **alertness** by measuring simple reaction time to 32 visual stimuli expressed in milliseconds. Subjects have to press a key as soon as a rectangle appears on the screen. The interval between two stimuli is variable in order to induce uncertainty about the timing of the appearance of the next stimulus. Mean reaction time and standard deviation of the reaction time were obtained for the dominant and non-dominant hand.

4. ‘GoNoGo’

This task is a measure of **response inhibition** and inattention. A key has to be pressed when a ‘go’-signal (a complete square) is presented. When a ‘no-go’-signal (an incomplete square) is presented, this prepotent response has to be inhibited. We used a balanced design with 24 ‘go’-signals and 24 ‘no-go’-signals, randomly presented. Mean reaction time of the hits, number of missed targets and number of false alarms were obtained. Response inhibition was measured by the percentage of false alarms.

5. ‘Memory Search Objects 2 keys’

This **divided attention** task measures **speed and accuracy of memory search processes**. An image of a house with four animals in the windows and the door is presented. Subjects have to press the yes-key when the house contains an animal from the memory set, and to press a no-key when this is not the case. The animals change positions in each trial. The task consists of two parts and memory load is increased with target set size rising from one animal in part one to two animals in part two. Divided attention is needed because all four stimuli are relevant and the subject has to divide the attention over the field of stimuli in order to search for animals from the target set. The reaction time for hits (RT hits) and for correct rejections (RT CR) were measured separately in part 1 and part 2, together with the number and percentage of missed targets (P-MI) and of false alarms on non-targets (P-FA). Also, the total percentage of errors was calculated as (P-MI_part1_ + *P*-FA_part1_ + *P*-MI_part2_ + *P*-FA_part2_)/4. The speed and accuracy of memory search processes were measured by calculating the increase in reaction time and error rate during higher memory load. A new variable Load[RT] was constructed as an index of the memory search rate, by calculating ((RT hits + RT CR)_part2_ – (RT hits + RT CR)_part1_)/2. Similarly, a new variable Load[Acc] was constructed as an index of the effect of increasing memory load on accuracy, calculated as ((P-MI + *P*-FA)_part2_ – (P-MI + *P*-FA)_part1_)/2.

6. ‘Focused Attention Objects 2 keys’

This task is a measure of **selective attention**. Four pieces of fruit are presented in a fruit basket, of which two pieces are located at the vertical axis (top and bottom) and two pieces at the horizontal axis (left and right). Subjects have to press the yes-key if the target fruit is presented at one of the two relevant locations, i.e. the left or right location of the horizontal axis. A no-response is required if the target fruit is shown at an irrelevant location (at the top or the bottom of the vertical axis) or if the target fruit is absent altogether. The three signal types (28 targets, 14 irrelevant targets, 14 non-targets) were presented in a random order. The reaction times for hits (RT hits), correct rejection of irrelevant targets (RT CR [irrelevant target]) and correct rejection of non-targets (RT CR [non-target]) were obtained, together with the number and percentage of missed targets (P-MI), false alarms on irrelevant targets (P-FA [irrelevant target]) and false alarms on non-targets (P-FA [non-target]). Focused attention is studied by examining the reaction time to targets presented on the irrelevant axis, since an attention shift to these targets illustrates a disruption of focused attention. The difference between the mean RT CR [irrelevant target] and RT CR [non-target] can be interpreted as a measure of the size of the distraction effect on RT. The difference between P-FA [irrelevant target] and P-FA [non-target] can be interpreted as a measure of the size of the distraction effect on accuracy. The accuracy of task performance is measured as the percentage of total errors, calculated as (2 x P-MI + *P*-FA [irrelevant target] + *P*-FA [non-target])/4. The mean reaction time gives an indication of overall processing speed.

Four subtasks from the Children's Memory Scale (CMS) were used to evaluate different aspects of memory [8].

7. ‘Numbers’

This task is a measure of the **verbal memory span** (repeating numbers forward) and **verbal working memory** (repeating numbers backward). Raw scores range from 0 to 16 (numbers forward) and from 0 to 14 (numbers backward), with higher scores indicating better performance.

8. ‘Picture Locations’

The **visuospatial memory span** is measured as the proportion of correctly recalled picture locations. The number of picture locations is gradually increased during the task, ranging from one to five pictures. Raw scores range from 0 to 30, with higher scores indicating better performance.

9. ‘Dot Locations’

This task is a measure of **visuospatial learning, short- and long-term visuospatial memory**. Subjects have to learn and recall the location of six blue dots. Three learning trials are offered. Next, subjects have to learn and recall a new pattern with six red dots in one trial. Immediate recall is measured as the proportion of correctly recalled blue dot locations after the interference of the red dots. Delayed recall is measured after 20 min of attention tasks of the ANT. Raw scores range from 0 to 6 for both the immediate and delayed recall phase, with higher scores indicating better performance.

10. ‘Faces’

This task is a measure of **short- and long-term memory for faces**. In the learning phase, subjects are presented 12 target faces. Next, 36 target and non-target faces are presented in a random order and the subject has to decide whether the face is a target or a non-target. The proportion of correctly recalled faces is a measure of immediate recall. After 20 min of attention tasks of the ANT (delayed recall), subjects are shown another series of 36 target and non-target faces presented in a random order and the subject has to decide again whether the face is a target or a non-target. Raw scores range from 0 to 36 for both the immediate and delayed recall phase, with higher scores indicating better performance.

Behavior questionnaire

The parents were asked to fill out a questionnaire on the incidence of **behavior problems** (Child Behavior Checklist, CBCL). [10] The items measure a range of emotional and behavioral problems on a three point Likert scale (0 = ‘not true’, 1 = ‘somewhat or sometimes true’, or 2 = ‘very true or often true’). The questionnaire consists of two empirically derived broadband scales (internalizing and externalizing problems) and several subscales. The total score of all problems results in the overall scale ‘total problems’. Raw scores are converted into standardized T-scores (mean 50, standard deviation 10), using computerized software provided by the developers of the questionnaire, which enables to control for gender, age and country.

### 2.3 Description of electrocardiography and echocardiographic evaluation

Cardiac evaluation included a 12-lead electrocardiography (ECG) and a full echocardiographic evaluation looking for structural and functional parameters. Heart rate and rhythm, blood pressure, PR interval, QRS duration, QT duration, and QT corrected for the heart rate were measured by the same observer. A functional pediatric echocardiogram was performed according to the standards of the American Society of Echocardiography guidelines [1] on a Vivid E-9 scanner (GE Ultrasound, USA). Cardiac dimensions were measured from a short-axis M-mode tracing just distal to the mitral valve leaflets. Left ventricular systolic function was assessed by calculating ejection fraction (EF) and shortening fraction (FS) from the M-mode tracing. Diastolic LV function was assessed by measuring pulsed Doppler traces of the mitral inflow, pulmonary venous flow and pulsed tissue Doppler traces from the mitral annulus according to ASE and EACVI recommendations [12]. Peak early peak velocity flow (E), deceleration time, A-velocity during atrial contraction (A) and E/A-ratio were calculated.

TDI and speckle-tracking analysis:

Offline analyze was performed for Tissue Doppler imaging (TDI) and speckle-tracking analysis by two independent observers using the EchoPac version 7.0 (GE Ultrasound, US) as our group previously validated the high reproducibility of TDI and speckle-tracking longitudinal strain with low intra- and interobserver variability [13, 14]. Two dimensional (2D) Grayscale images for offline speckle-tracking analysis are acquired at frame rates of 50 to 90 frames/s. Analysis was performed as previously described using the 2-D strain method (Echopac, GE Ultrasound, US) [15]. LV circumferential strain was measured from the parasternal short-axis view at mid-ventricular level (showing both papillary muscles). Peak systolic circumferential strain was measured in the anterior, anterolateral inferolateral, inferior, inferoseptal and anteroseptal segments. Mean circumferential strain was calculated as the mean value of the six segmental measurements. Longitudinal strain was measured form apical four-chamber view in the basal, mid and apical septal and lateral wall segments. Tracking was automatically performed, and the analysis is accepted after visual inspection. Electrocardiographic measurements were analyzed and compared with normal values in childhood and adolescence published by Dickinson.^5^ All cardiac measurements were compared to the measurements in a 1:1 matched control group for age (+/- 12 months) and gender.

### 2.4 Questionnaire on general health and education of child

Questionnaire regarding general health of the child

1. Which type of education is your child following? Are there any problems at school?

……………………………………………………………………………………………

2. Have there been changes in your family situation since the birth of ………… (Name child)?

……………………………………………………………………………………………
……………………………………………………………………………………………
……………………………………………………………………………………………

3. Which language(s) is/are spoken at home?

……………………………………………………………………………………………

4. In which language does your child follow education at school?

……………………………………………………………………………………………

5. Did your child suffer from a disease?

Did or does your child suffer from one of the following diseases?
*If yes, please specify the disease and age at diagnosis*
Respiratory disorder: yes/no…………………………………………………………..
Disorder of the eyes: yes/no…………………………………………………………
Does your child wear glasses: yes/no
Disorder of the ears: yes/no…………………………………………………………
Did your child have a hearing test (audiometry): yes/no
Was the test normal/abnormal, and if abnormal, which problems have been found? ……………………………………………………………………………
Disorder of the heart and vessels: yes/no……………………………………………
Disorder of the gastro-intestinal system: yes/no………………………………..……
Disorder of the bones and joints: yes/no……………………………….……………
Disorder of the kidneys and urinary tract: yes/no……………………………………
Hormonal disorder: yes/no…………….………………………………………………
Skin-or hair disorder: yes/no..…………………………………………………………
Allergy: yes/no………………………………………………………….…………….
Disorder of the genital tract: yes/no………..…………………………………………
Congenital problems (internally or externally): yes/no………………………………
Hereditary disorders: yes/no………………………………………...........................…
Other serious health problems: yes/no………………………………………………
Motor problems: yes/no………………………………………………………………
Is there damage to the central nervous system? Yes/no ……………………………..
Are there any teeth problems? Yes/no ……………………………………………….
Other problems: yes/no ..…………………………………………………….……….

6. Sexual development: How old was your child at:

* Boy
Start pubis/ armpit pilosity: …………………….
Changes of the voice: …………………………….
Face pilosity (barb/moustache):………………….
* Girl
First menstruation: ……………………………..
Development of the breast: ……………………………....
Start pubis/ armpit pilosity: …………………………..

7. Has your child been operated? If yes, please specify the type, reason and date of surgery.

…………………………………………………………………………………………..
……………………………………………………………………………………………
……………………………………………………………………………………………

8. Does/did your child take medication chronically? If yes, for what reason?

……………………………………………………………………………………………
……………………………………………………………………………………………

9. Does your child need another special treatment or supportive care? If yes, for what reason?

……………………………………………………………………………………………
……………………………………………………………………………………………
…………………………………………………………………………………………..

10. Does your child play sports? If yes, please clarify which sport and how many hours a week?

……………………………………………………………………………………………
……………………………………………………………………………………………

11. Does your child have other hobby's?

…………………………………………………………………………………………..
……………………………………………………………………………………………

12. Do you have information about the growth of your child at different ages?

**Table:** 

Date of measurement	Height (cm)	Weight (kg)	Head circumference (cm)
			
			
			
			
			
			

13. Remarks:

……………………………………………………………………………………………
……………………………………………………………………………………………
……………………………………………………………………………………………
……………………………………………………………………………………………………
Thanks a lot for your cooperation!

### 2.5 Additional information on the statistical analysis

In our study protocol, we specified to analyze between-group differences in neurocognitive outcomes by means of ANOVA. However, we did not expect that the education level of the parents would be different between the study and control group. Although not statistically significant, there are differences in parental education levels (i.e., parents of children in the control group are in general higher educated than parents of children in the study group) which might impact on the neurocognitive outcomes. Therefore, we chose to add parental education levels as a covariate in the analysis. We also considered other possible confounding factors. As we matched for gender, gestational age at birth, test age, country, and language of the tests, the groups were equal considering these variables and therefore these variables may not have a differential impact between the groups. We also found that the groups were comparable in terms of maternal age, smoking and alcohol use during pregnancy, fertility treatment, and bilingual education of the child. Therefore, we did not include them as a covariate in the analysis.

We used ANOVA for the cardiac outcomes. We did not include covariates as the groups had already been matched for gender and age and no other variables that were measured were considered as potentially confounding factors.

Furthermore, we described in our study protocol that we intended to assess the relationship between intelligence quotient and gestational age at birth by linear (ordinary least squares) regression and that the effect size would be estimated with the omega2 measure of explained variance. We planned to add age and sex as covariates, together with a random effect for country. We did investigate the relationship between intelligence quotient (IQ) scores and gestational age at birth, but we used correlation analysis instead of regression analysis. When no covariates are included, correlation coefficients and Beta coefficients (regression analysis) are likely to be very close to each other. Note that this is likely to be truer for Pearson correlations than for Spearman correlations. Country could not be included as a random effect, as the number of countries (only 4) and the number of participants per country (for some countries) were too small for multilevel analysis. At the beginning of the study, we hypothesized that more countries from the International Network on Cancer, Infertility and Pregnancy would join this part of the study.

Partial eta squared was used as a measure of effect size in ANCOVA. Partial eta squared looks at the proportion of variance that a variable explains that is not explained by other variables in the analysis. A partial eta squared value of 0.01 can be interpreted as a small effect, 0.09 as a medium effect and 0.25 as a large effect.

## Ethics Statement

Ethical approval was obtained by each institution and the parents of each child provided written informed consent to participate. The full study protocol is available at http://www.cancerinpregnancy.org/study-protocols.

## Declaration of Competing Interest

The authors declare that they have no known competing financial interests or personal relationships which have, or could be perceived to have, influenced the work reported in this article.

## Appendix Supplementary materials

Image, application 1
