# Supplementary material for: Data describing child development at 6 years after maternal cancer diagnosis and treatment during pregnancy
Source: Data Brief. 2020 Aug 21;32:106209. doi: 10.1016/j.dib.2020.106209 (PMC7479323; doi:10.1016/j.dib.2020.106209)
Supplement: Supplementary file 1 [file mmc1.docx]

**Supplementary appendix**

This appendix has been provided by the authors to give readers additional information about their work.

**Data describing child development at 6 years after maternal cancer diagnosis and treatment during pregnancy**

**Table of contents**

[1. Results 3](#_Toc38351577)

[1.1 Overview of registered dosages received per drug (eTable 1) 3](#_Toc38351580)

[1.2 Overview of radiation exposure in 13 patients (including 1 twin pregnancy) and the gestational period of exposure (eTable 2) 4](#_Toc38351581)

[1.3 Diagnostic and staging examinations performed during pregnancy, registered in 97 patients (eTable 3) 5](#_Toc38351582)

[1.4 Registered surgical procedures executed between 2.7 and 32.4 weeks gestational age in 71 patients (eTable 4) 6](#_Toc38351583)

[1.5 Overview of supportive and other drugs during pregnancy as registered in 92 patients (eTable 5) 7](#_Toc38351584)

[1.6 Overview of aprepitant use during pregnancy and child outcomes (eTable 6) 10](#_Toc38351585)

[1.7 Smoking during pregnancy for study and control children (eTable 7) 11](#_Toc38351586)

[1.8 Alcohol use during pregnancy for study and control children (eTable 8) 11](#_Toc38351587)

[1.9 Fertility treatment to achieve this pregnancy for study and control children (eTable 9) 12](#_Toc38351588)

[1.10 Bilingual education from birth to 6 years for study and control children (eTable 10) 12](#_Toc38351589)

[1.11 Registered congenital malformations in 132 children per cancer treatment modality (eTable 11) 13](#_Toc38351590)

[1.12 Parameters for the calculation of birth weight percentiles with customized growth curves in singleton pregnancies (eTable 12) 14](#_Toc38351591)

[1.13 Biometric curves (weight, height and head circumference) at 6 years for study and control children (males and females separate) (eFigure 1) 15](#_Toc38351592)

[1.14 Biometric curves (weight and height) at 6 years for study and control children born small for gestational age (males and females separate) (eFigure 2) 18](#_Toc38351597)

[1.15 Intelligence outcomes in children from the cancer in pregnancy group compared to matched controls using ANCOVA with parental education levels as covariates (eTable 13) 20](#_Toc38351602)

[1.16 Uncorrected intelligence outcomes in children from the cancer in pregnancy group and the control group presented in boxplots (eFigure 3) 21](#_Toc38351603)

[1.17 Intelligence outcomes in chemotherapy-exposed children compared to matched controls using ANCOVA with parental education levels as covariates (eTable 14) 23](#_Toc38351604)

[1.18 Full Scale IQ in relation to the estimated fetal dose of radiation (expressed in milligrays) for 14 children exposed to radiotherapy during pregnancy (eFigure 4) 24](#_Toc38351605)

[1.19 Verbal IQ in children exposed to anthracyclines, FAC/FEC chemotherapy, taxanes or platin-based treatments compared to matched controls using ANCOVA with parental education levels as covariates (eTable 15) 25](#_Toc38351606)

[1.20 Verbal IQ in study children whose mother died and those with surviving mothers compared to matched controls using ANCOVA with parental education levels as covariates (eTable 16) 27](#_Toc38351607)

[1.21 Memory outcomes in children from the cancer in pregnancy group compared to matched controls using ANCOVA with parental education levels as covariates (eTable 17) 28](#_Toc38351608)

[1.22 Memory outcomes in chemotherapy-exposed children compared to matched controls using ANCOVA with parental education levels as covariates (eTable 18) 29](#_Toc38351609)

[1.23 Attention outcomes in children from the cancer in pregnancy group compared to matched controls using ANCOVA with parental education levels as covariates (eTable 19) 30](#_Toc38351610)

[1.24 Attention outcomes in chemotherapy-exposed children compared to matched controls using ANCOVA with parental education levels as covariates (eTable 20) 31](#_Toc38351611)

[1.25 Behavior problems in children from the cancer in pregnancy group compared to matched controls using ANCOVA with parental education levels as covariates (eTable 21) 32](#_Toc38351612)

[1.26 Behavior problems in chemotherapy-exposed children compared to matched controls using ANCOVA with parental education levels as covariates (eTable 22) 32](#_Toc38351613)

[1.27 Behavior problems in study children whose mother died and those with surviving mothers compared to matched controls using ANCOVA with parental education levels as covariates (eTable 23) 33](#_Toc38351614)

[1.28 Echocardiographic measurements, pulsed tissue Doppler imaging (TDI) and speckle-tracking measurements in children exposed to anthracyclines compared to matched controls using ANOVA (eTable 24) 34](#_Toc38351615)

[1.29 General health problems of study and control children, registered by the parents (eTable 25) 36](#_Toc38351616)

[1.30 Hearing loss in children prenatally exposed to cisplatin (eTable 26) 38](#_Toc38351617)

[1.31 Abnormalities observed during history taking and general pediatric and clinical neurological examination in 103 study children undergoing examination (eTable 27) 39](#_Toc38351619)

# 1. Results

## 1.1 Overview of registered dosages received per drug (eTable 1)

| **Anti-cancer agent** | **N patients*** | **Cumulative dosage (mg/m²): median (range)** |
| --- | --- | --- |
| Doxorubicin | 28/32 | 180 (50-360) |
| Epirubicin | 34/37 | 300 (70-600) |
| Daunorubicin | 2/2 | 45 |
| Idarubicin | 2/2 | 30 (24-36) |
| Cyclophosphamide | 49/56 | 1800 (600-4500) |
| 5-Fluorouracil | 30/34 | 1500 (600-3000) |
| Docetaxel | 9/10 | 300 (100-400) |
| Paclitaxel | 7/9 | 350 (140-1050) |
| Cisplatin | 10/13 | 300 (60-450) |
| Carboplatin | 4/5 | 1235 (210-2000) |
| Vincristine | 5/7 | 4 (1-9) |
| Bleomycine | 5/5 | 50 (40-80) |
| Dacarbazine | 5/5 | 1850 (800-3000) |
| Vinblastine | 5/5 | 30 (24-48) |
| Cytarabine | 3/4 | 200 (3-300) |
| Rituximab | 1/2 | 1500 |
| Methotrexate | 2/3 | 27.5 (15-40) |
| Mitoxantrone | 1/1 | 10 |
| 6-Thioguanine | 1/1 | 120 |
| Amsacrin | 0/1 | Not reported |
| Temozolomide | 1/1 | 3000 |
| *Number of patients with registered dosages / Total number of patients receiving this type of anti-cancer agent. | | |
|  |  |  |

## 1.2 Overview of radiation exposure in 13 patients (including 1 twin pregnancy) and the gestational period of exposure (eTable 2)

| **Patient** | **Cancer type** | **Radiation Field** | **GA (w)** | **Maternal Dose (Gy)** | **Estimated Fetal Dose (mGy)** |
| --- | --- | --- | --- | --- | --- |
| 1 | Tongue | Head and neck | 17-21 | 60 | 10 |
| 2 | Thyroid | Head and neck | 11-17 | 46 | 66 |
| 3 | NHL | Head | 28 | 33 | 34 |
| 4 | Brain | Head | 16-19 | 54 | 42 |
| 5 | AML | Left eye | 20-22 | 20 | 15 |
| 6 (twin) | Tongue | Head and neck | 15-17 | 60 | 10 |
| 7 | Breast | Breast | 10-15 | 60 | 191 |
| 8 | Breast | Thoracic wall | 19-23 | 46 | 131.7 |
| 9 | Soft tissue liposarcoma (tigh) | Left tigh | 19 | 50 (brachytherapy) | 2.5 |
| 10 | Tongue | Head and neck | 27-34 | 66 | 46 |
| 11 | Breast | Breast | 14-21 | 70 | 153 |
| 12 | Breast | Chest wall | 21-27 | 66 | 52 |
| 13 | Tongue | Neck | 7-14 | 66 | 100 |
| Abbreviations: GA, gestational age; w, weeks; Gy, Gray; mGy, milliGray; NHL, Non-Hodgkin’s disease; AML, acute myeloid leukemia | | | | | |

The dose program “Peridose” developed by van der Giessen was used to estimate the fetal radiation dose.[1]

## 1.3 Diagnostic and staging examinations performed during pregnancy, registered in 97 patients (eTable 3)

| **Examination** | **Number*** | **Gestational age: median (range)** |
| --- | --- | --- |
| Ultrasound breast + axilla | 56 | 19.0 (2.0-37.6) |
| Echocardiography | 21 | 25.6 (17.1 – 34.6) |
| Ultrasound liver | 34 | 19.8 (2.1 – 35.7) |
| Ultrasound gall bladder | 1 | 24.9 |
| Ultrasound abdomen | 24 | 21.4 (4.7 – 32.1) |
| Ultrasound neck (+ thyroid) | 13 | 21.1 (2.6 – 35.1) |
| Ultrasound limbs | 2 | 12.4 – 25.4 |
| Ultrasound pelvis | 2 | 28.0 – 33.0 |
| Mammography | 18 | 20.4 (2.0 – 36.4) |
| X-Ray thorax | 51 | 19.4 (2.1 – 36.0) |
| CT thorax | 13 | 25.3 (13.7 – 27.7) |
| CT abdomen | 4 | 20.0 (15.4 – 27.1) |
| CT neck | 1 | 19.4 |
| CT brain | 3 | 14.9 (14.4 – 19.6) |
| MRI whole body (+ thorax-abdomen) | 7 | 26.1 (12.7 – 29.7) |
| MRI thorax | 6 | 16.5 (2.3 – 28.3) |
| MRI abdomen (+ liver) | 12 | 27.2 (5.1 – 29.7) |
| MRI pelvis | 14 | 19.6 (4.6 – 33.7) |
| MRI brain | 12 | 23.3 (13.1 – 31.7) |
| MRI spine | 5 | 20.9 (12.1 – 28.7) |
| MRI head + neck | 3 | 10.1 (8.6 – 30.3) |
| MRI breast | 2 | 32.7 (32.1 – 33.3) |
| MRI limbs | 2 | 13.9 (12.9 – 14.9) |
| Nuclear bone scan | 9 | 20.6 (2.4 – 25.7) |
| PET/CT | 3 | 10.3 (2.9 – 16.0) |
| Gastro(duodeno)scopy | 3 | 12.0 (10.1 – 29.6) |
| Colo + Recto-scopy | 1 | 19.7 |
| Bone marrow biopsy | 10 | 16.9 (14.6 – 32.4) |
| Cystoscopy | 3 | 17.9 (6.7 – 30.4) |
| Ophtalmoscopy | 1 | 28.6 |
| Colposcopy (pap-smear and biopsy) | 5 | 16.7 (6.9 – 31.4) |
| Vitrectomy | 1 | 18.0 |
| * Number of all examinations registered, most of the patients underwent more than one of the same examination or a combination of the examinations.  Abbreviations: CT, Computed Tomography; MRI, Magnetic Resonance Imaging; PET, Positron Emission Tomography | | |
|  |  |  |
|  |  |  |

## 1.4 Registered surgical procedures executed between 2.7 and 32.4 weeks gestational age in 71 patients (eTable 4)

| **Surgical procedure** | **N** |  |
| --- | --- | --- |
| Breast lumpectomy | 2 |  |
| Mastectomy | 2 |  |
| SLNB | 2 |  |
| Breast lumpectomy + SLNB | 5 |  |
| Breast lumpectomy + ALND | 9 |  |
| Mastectomy + SLNB | 3 |  |
| Mastectomy + ALND | 17 |  |
| Mastectomy + ALND + reconstruction | 1 |  |
| Breast lumpectomy + SLNB + ALND | 4 |  |
| Laparoscopic adnexectomy | 4 |  |
| Laparoscopic cystectomy | 1 |  |
| Partial colectomy | 1 |  |
| Omentectomy/ appendectomy/ diaphram stripping by laparotomy | 1 |  |
| Omentectomy/ appendectomy/ biopsies by laparoscopy | 2 |  |
| Omentectomy/ adnexectomy/ biopsies by laparoscopy | 2 |  |
| Examination under general anaesthesia + cystoscopy | 4 |  |
| Examination under general anaesthesia + cystoscopy + pelvic lymphadenectomy | 1 |  |
| Thyroidectomy + total lymph node resection of the neck | 1 |  |
| Total lymph node resection of the neck | 1 |  |
| Endoscopic intracranial surgery | 1 |  |
| Craniotomy | 2 |  |
| Resection skin lesion | 1 |  |
| Resection muscular mass | 1 |  |
| Partial glossectomy / lymph node resection of the neck / reconstruction of the tongue | 1 |  |
| Resection tongue lesion / lymph node resection of the neck | 1 |  |
| Partial nephrectomy / total para-aortic and aortocaval lymph node resection | 1 |  |
| LLETZ | 2 |  |
| Pelvic lymphadenectomy | 2 |  |
| Diagnostic laparoscopy followed by laparotomic resection of lymphoma and suturing of gastric perforation | 1 |  |
| Abbreviations: SLNB, sentinel lymph node biopsy; ALND, axillary lymph node dissection; LLETZ, large loop excision of the transformation zone | | |
|  |  |  |

## 1.5 Overview of supportive and other drugs during pregnancy as registered in 92 patients (eTable 5)

|  | **Drug** | **N*** | **Dose range** |
| --- | --- | --- | --- |
| **Antibiotics** | amoxicillin | 8 | 500-1000mg (3-4x/day) |
|  | amoxicillin-clavulanate | 11 | 625-1200mg (1-3x/day) |
|  | cefuroxim | 1 | 75-500mg (3x/day) |
|  | ceftriaxone | 1 | 4x 4g |
|  | ceftazidime | 1 | unknown |
|  | cefepime | 1 | 2mg |
|  | piperacillin/tazobactam | 1 | 1g (4x/day) |
|  | erythromycin | 1 | 500mg |
|  | flucloxacilline | 2 | 500mg (4x/day) |
|  | oxacilline | 1 | 1g |
|  | nitrofurantoin | 3 | 100mg/day |
|  | antibiotics unspecified | 1 | unknown |
| **Antiemetics** | methylprednisolone/prednison | 25 | 16-125mg |
|  | dexamethasone | 22 | 3-14mg (1-3x/day) |
|  | ondansetron | 23 | 8mg (1-3x/day) / 125mg IV |
|  | tropisetron | 10 | 0.1-5mg |
|  | granisetron | 6 | unknown |
|  | aprepitant* | 6 | 80-125mg (see next table) |
|  | alizapride | 15 | 50mg (1-6x/day) |
|  | metoclopramide | 12 | 10-20mg (1-4x/day) |
|  | meclozine | 1 | unknown |
|  | H_2_ antihistamines (1 not specified) | 16 |  |
|  | clemastine | 4 | 2mg |
|  | ranitidine | 9 | 50-150mg |
|  | chlorpheniramine | 2 | 10mg |
| **Analgetics** | paracetamol | 11 | 800mg-2g |
|  | piritramide | 2 | 2mg |
|  | tramadol | 1 | 100mg |
|  | NSAID | 1 | 75mg |
| **Colony Stimulating Factor** | pegfilgrastim | 4 | 6mg |
|  | lenograstim | 1 | 263µg |
| **Lung Maturation** | betamethasone | 24 | 1-2x 12mg |
| **Tocolytics** | beta-2 mimetics | 1 | unknown |
|  | atosiban | 3 | unknown |
|  | indometacin | 1 | unknown |
|  | calciumantagonists (nifedipine) | 4 | 10mg |
| **Miscellaneous** | proton-pump inhibitors | 3 |  |
|  | omeprazole | 2 | 20mg |
|  | pantoprazole | 1 | 2x 40mg |
|  | benzodiazepines or related | 9 |  |
|  | prazepam | 1 | unknown |
|  | alprazolam | 3 | 0.25-0.5mg |
|  | lormetazepam | 2 | 1mg |
|  | diazepam | 1 | unknown |
|  | zolpidem | 1 | unknown |
|  | temazepam | 1 | unknown |
|  | low molecular weight heparines (enoxaparine, nadroparine) | 12 | 20-40mg |
|  | acetylsalicylic acid | 1 | 80mg |
|  | laxatives | 2 | 1-3 bags per day |
|  | antacids | 2 | 1g (3-4x/day) |
|  | antihypotensive agents (norepinephrine, etilefrine) | 2 | unknown |
|  | antihypertensive agents (methyldopa) | 1 | 500mg (3x/day) |
|  | vitamins, minerals (pyridoxine, magnesium sulphate, folic acid, iron, calcium carbonate) | 36 | unknown |
|  | anti-Rho immunoglobulines (D) | 2 | unknown |
|  | L-thyroxine | 4 | 100-200µg |
|  | progesterone | 4 | 200mg (3x/day) |
|  | digoxin | 1 | 2x 0.5mg |
|  | flecainide acetate | 1 | 2x 100mg |
|  | H_1_ antihistamines (cetirizine) | 1 | unknown |
|  | insulin | 1 | 10 units / day |
|  | propylthiouracil | 1 | unknown |
|  | selective serotonin reuptake inhibitor (paroxetine) | 1 | 20mg |
|  | homeopathics | 1 | unknown |
|  | venoactive agents | 2 |  |
|  | daflon | 1 | 200-450 (2x/day) |
|  | spasfon | 1 | unknown |
|  | ursodeoxycholic acid | 1 | unknown |
|  | anti-epileptic agents | 2 |  |
|  | lamotrigine | 1 | 100mg (2x/day) – 125mg |
|  | valproic acid | 1 | 300mg |
|  | proteolytic enzymes | 2 | unknown |
|  | diuretics (furosemide) | 1 | 20mg |
|  | immunosuppressive agents (azathioprine) | 1 | unknown |
| Data were not available for all patients because registration in the medical records is incomplete.  *Data on aprepitant use during pregnancy are scarce in the literature, therefore we specified the use of aprepitant in our patient sample in the next table. | | |  |

## 1.6 Overview of aprepitant use during pregnancy and child outcomes (eTable 6)

| Patient | Chemotherapeutic scheme | Gestational age at chemotherapy (weeks) | Dose of aprepitant | Child outcome at the age of 6 years |
| --- | --- | --- | --- | --- |
| 1 | 3 cycles of FEC | 25.6  28.6  31.6 | 125mg day 1 after FEC chemo,  80mg day 2 and 3 after FEC chemo | FSIQ = 133, memory and attention skills average to above average.  Health problems: reduced vision, allergy to penicillin. |
| 2 | 3 cycles of AC | 25.4  28.1  31.3 | During chemotherapy, dose unknown | FSIQ = 115, memory and attention skills average to above average.  No health problems. |
| 3 | 4 cycles of EC | 14.0  17.0  20.0  23.0 | 125mg (not specified when it was given) | FSIQ = 101, memory and attention skills average.  No health problems. |
| 4 | 3 cycles of FEC  3 cycles of docetaxel | 17.7  20.7  23.6  26.6  29.7  32.7 | 125mg + 80 mg + 80mg before chemotherapy (not specified whether it was only during FEC or also during docetaxel) | FSIQ = 87, memory span below average, other memory skills average, attention skills below average.  Health problems: Wolff-Parkinson-White syndrome. |
| 5 | 3 cycles of FEC | 29.6  32.4  35.4 | Unknown | FSIQ = 104, memory and attention skills average to above average.  No health problems. |
| 6 | 6 cycles of cisplatin + paclitaxel  2 cycles of paclitaxel only | 20.0  21.0  22.0  23.0  24.0  25.0  29.0  30.0 | 125mg day 1, 80mg day 2 and 3 (not specified whether it was only during cisplatin + paclitaxel or also during paclitaxel only) | FSIQ = 80, memory skills below average to average, attention skills below average.  Health problems: allergy to food additives |

Abbreviations: AC, doxorubicin, cyclophosphamide; (F)EC, 5-fluorouracil, epirubicin, cyclophosphamide; FSIQ, Full Scale IQ score

Note: it is possible that more patients were exposed to aprepitant as registration in the medical records is incomplete.

## 1.7 Smoking during pregnancy for study and control children (eTable 7)

|  | N of mothers smoked during pregnancy (%) | Median number of cigarettes per week (range) |
| --- | --- | --- |
| Cancer in pregnancy group | 6 (5.4 %) | 15 (5-60) |
| Control group | 11 (8.9 %) | 10 (0.5-70) |

Information on smoking during pregnancy was available for 112/132 study children and 123/132 control children.

## 1.8 Alcohol use during pregnancy for study and control children (eTable 8)

|  | N of mothers drinking alcohol during pregnancy (%) | Reported number of consumptions during pregnancy |
| --- | --- | --- |
| Cancer in pregnancy group | 3 (2.7 %) | 1-2 consumptions per week (N=3) |
| Control group | 9 (7.4 %) | Less than one per month (N=1)  Less than one per week (N=6)  1-2 consumptions per week (N=2) |

Information on alcohol use during pregnancy was available for 112/132 study children and 122/132 control children.

## 1.9 Fertility treatment to achieve this pregnancy for study and control children (eTable 9)

|  | N of mothers pregnant through fertility treatment (%) | Type of fertility treatment |
| --- | --- | --- |
| Cancer in pregnancy group | 14 (11.5 %) | Hormonal stimulation (N=4)  IUI (N=1)  IUI with donor sperm (N=1)  IVF (N=4)  ICSI (N=3)  Unknown (N=1) |
| Control group | 12 (10.1 %) | Hormonal stimulation (N=1)  IUI (N=3)  IVF (N=4)  ICSI (N=3)  Unknown (N=1) |

Information on the need of fertility treatment to achieve this pregnancy was available for 122/127 mothers of study children (including 5 twin pregnancies) and 119/126 mothers of control children (including 6 twin pregnancies).

Abbreviations: IUI = intra-uterine insemination, IVF = in vitro fertilization, ICSI = intracytoplasmatic sperm injection

## 1.10 Bilingual education from birth to 6 years for study and control children (eTable 10)

|  | N of children raised bilingual |
| --- | --- |
| Cancer in pregnancy group | 30 (23.8%) |
| Control group | 26 (20.8%) |

Children were considered to be raised bilingual if they were equally exposed to two languages at home or if at least half of the classes at school were taught in another language than the child’s mother tongue.

Information on bilingual education was available for 126/132 study children and 125/132 control children.

## 1.11 Registered congenital malformations in 132 children per cancer treatment modality (eTable 11)

| **Treatment during pregnancy** | **Minor malformation N=7, 5.3%**  **Major malformation* N=2, 1.5%** |
| --- | --- |
| Chemotherapy | Two little muscular ventricular septal defects (N=1)  Absent uvula, undescended testicles (N=1)  Accessory ear tag (N=1) |
| Chemotherapy + Surgery | Duplex pyelum (N=1)  Stenosis ani (N=1)  Ulnar polydactyly (N=1)  Naevus flammeus in groin (N=1)  *Anorectal atresia (N=1) |
| Chemotherapy + Radiotherapy | / |
| Chemotherapy + Surgery + Radiotherapy | / |
| Surgery | / |
| Radiotherapy | / |
| Surgery + Radiotherapy | / |
| No treatment | *Hip subluxation (N=1) |
| Malformations are defects of organs or body parts due to an intrinsically abnormal developmental process. In this process, a structure is not formed, is partially formed, or is formed in an abnormal fashion. Major malformations are those that have medical and/or social implications. A major malformation is defined as one that is incompatible with survival, such as anencephaly; or one requiring major surgery for correction, such as cleft palate or congenital heart disease; or one producing major dysfunction (e.g., mental retardation). Minor malformations have mostly cosmetic significance. They rarely are medically significant or require surgical intervention. They represent part of the normal variation in the general population. (Definition according to Eurocat; www.eurocat-network.eu) | |
|  |  |
|  |  |

## 1.12 Parameters for the calculation of birth weight percentiles with customized growth curves in singleton pregnancies (eTable 12)

|  | **Cancer in pregnancy group (N=121)** | | **Control group (N=119)** | |
| --- | --- | --- | --- | --- |
|  | **Median (range) / N (%)** | **Available N** | **Median (range) / N (%)** | **Available N** |
| **GA** (weeks) | 36.6 (27.4 – 40.7) | 121 | 36.1 (28.6 – 41.0) | 119 |
| **Birth weight** (g) | 2760 (720 – 4200) | 121 | 2760 (1025 – 4400) | 119 |
| **Gender**  Male  Female | 64 (52.9%)  57 (47.1%) | 121 | 64 (52.9%)  57 (47.1%) | 119 |
| **Maternal country of origin ***  Belgium  Netherlands  Italy  Czech Republic  Morocco  Turkey  Sub-Saharan Africa  Other | 68 (57.1%)  20 (16.8%)  11 (9.2%)  7 (5.9%)  5 (4.2%)  2 (1.7%)  3 (2.5%)  3 (2.5%) | 119 | 76 (65.5%)  21 (18.1%)  10 (8.6%)  4 (3.4%)  2 (1.7%)  0  1 (0.9%)  2 (1.7%) | 116 |
| **Maternal length** (cm) ** | 165 (154 – 182) | 105 | 168 (150 – 185) | 107 |
| **Maternal weight at booking** (kg) *** | 62 (47 – 120) | 106 | 63 (39 – 90) | 103 |
| **Parity**  Primiparae  Multiparae | 50 (42.0%)  69 (58.0%) | 119 | 57 (50.0%)  57 (50.0%) | 114 |

Birth weight percentiles were calculated considering the gestational age at birth, sex, country of origin, parity, and maternal length and weight when available (www.gestation.net, v8.0.2, 2018). Twins were excluded.

* For missing values Global Average is used.

** For missing values the software uses standard values according to country of origin (e.g., 168cm for Belgium, 170cm for the Netherlands, 164cm for Italy, 165cm for the Czech Republic, 165cm for Global Average).

*** For missing values the software uses standard values according to country of origin (e.g., 64kg for Belgium, 67kg for the Netherlands, 60kg for Italy, 64 kg for the Czech Republic, 64kg for Global Average).

## 1.13 Biometric curves (weight, height and head circumference) at 6 years for study and control children (males and females separate) (eFigure 1)

##

##

##

##

## 1.14 Biometric curves (weight and height) at 6 years for study and control children born small for gestational age (males and females separate) (eFigure 2)

##

## In the subgroup of SGA children, we observed a catch-up weight in 68.8% of study children (11/16, 2 unknown) versus in 71.4% of controls (5/7).

##

## In the subgroup of SGA children, we observed a catch-up height in 93.8% of study children (15/16, 2 unknown) versus in 85.7% of controls (6/7).

## 1.15 Intelligence outcomes in children from the cancer in pregnancy group compared to matched controls using ANCOVA with parental education levels as covariates (eTable 13)

| Measurement | No. | Cancer in pregnancy group (N=132) | | | | Control group (N=132) | | | | Type 3 test of fixed effects | | | |
| --- | --- | --- | --- | --- | --- | --- | --- | --- | --- | --- | --- | --- | --- |
|  |  | **Mean** | S.E. | 95% CI | | **Mean** | S.E. | 95% CI | | F | P value | Q value | Partial eta squared |
|  |  |  |  | Lower | Upper |  |  | Lower | Upper |  |  |  |  |
| Full Scale IQ | 251 | **98.9** | 1.9 | 95.2 | 102.6 | **103.0** | 2.1 | 98.9 | 107.0 | 4.96 | 0.03 | 0.16 | 0.020 |
| Verbal IQ | 236 | **98.1** | 1.9 | 94.5 | 101.8 | **104.4** | 2.0 | 100.4 | 108.4 | 12.22 | 0.001 | <0.001 | 0.051 |
| Performance IQ | 236 | **100.4** | 1.9 | 96.6 | 104.2 | **101.4** | 2.1 | 97.2 | 105.6 | 0.32 | 0.57 | 0.71 | 0.001 |
| Processing Speed | 203 | **98.8** | 2.4 | 94.1 | 103.6 | **101.4** | 2.6 | 96.3 | 106.6 | 1.30 | 0.26 | 0.42 | 0.007 |

Abbreviations: CI, confidence interval; S.E., standard error of the mean

Results are expressed as standardized IQ-scores (M=100, SD=15). Higher numbers indicate better performance. Estimated marginal means are presented.

Raw P values are presented. The Q value represents the false discovery rate adjusted P value.

## 1.16 Uncorrected intelligence outcomes in children from the cancer in pregnancy group and the control group presented in boxplots (eFigure 3)


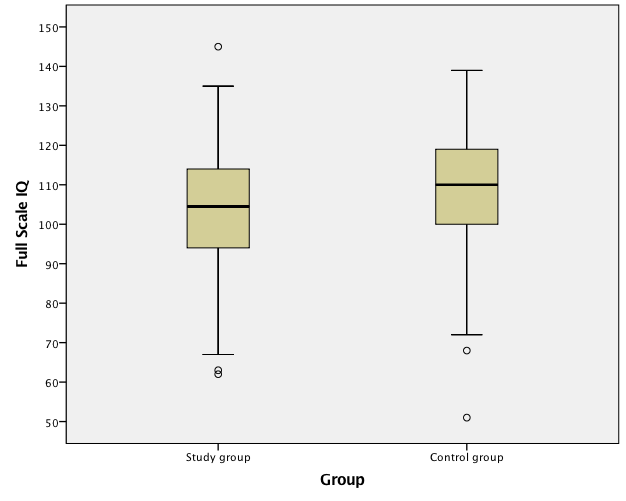


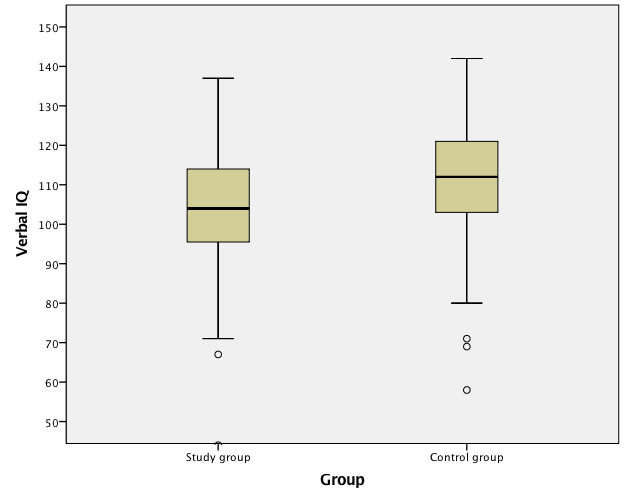


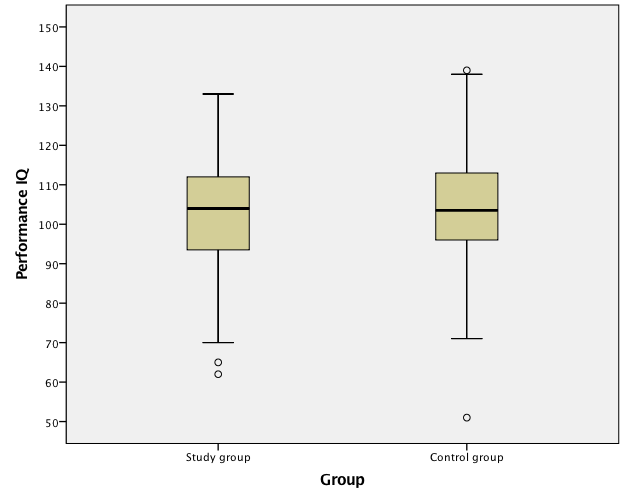


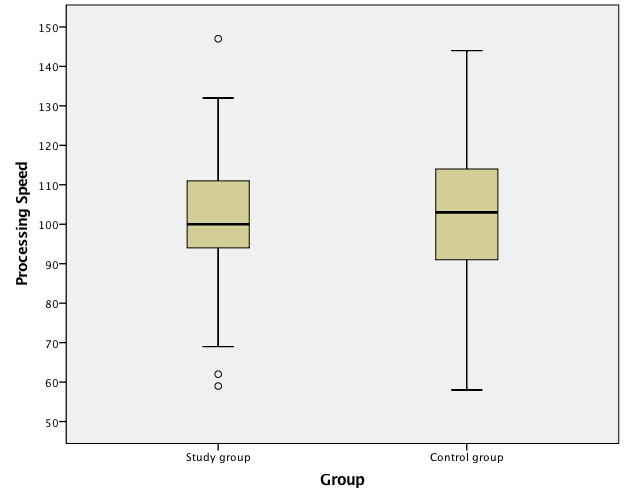


Note: Uncorrected intelligence scores are presented in the boxplots. Therefore, these scores may deviate from the scores presented in the previous table, as the scores in Table S15 are corrected for parental education levels.

## 1.17 Intelligence outcomes in chemotherapy-exposed children compared to matched controls using ANCOVA with parental education levels as covariates (eTable 14)

| Measurement | No. | Chemotherapy group (N=97) | | | | Control group (N=97) | | | | Type 3 test of fixed effects | | | |
| --- | --- | --- | --- | --- | --- | --- | --- | --- | --- | --- | --- | --- | --- |
|  |  | **Mean** | S.E. | 95% CI | | **Mean** | S.E. | 95% CI | | F | P value | Q value | Partial eta squared |
|  |  |  |  | Lower | Upper |  |  | Lower | Upper |  |  |  |  |
| Full Scale IQ | 184 | **101.4** | 3.9 | 93.7 | 109.1 | **106.0** | 4.1 | 98.0 | 114.0 | 4.27 | 0.04 | 0.18 | 0.024 |
| Verbal IQ | 169 | **101.7** | 3.7 | 94.3 | 109.0 | **108.5** | 3.9 | 100.8 | 116.1 | 9.65 | 0.002 | 0.04 | 0.057 |
| Performance IQ | 169 | **101.0** | 3.9 | 93.2 | 108.8 | **102.4** | 4.1 | 94.3 | 110.5 | 0.35 | 0.56 | 0.64 | 0.002 |
| Processing Speed | 142 | **101.1** | 4.4 | 92.4 | 109.8 | **103.9** | 4.6 | 94.7 | 113.0 | 0.97 | 0.33 | 0.49 | 0.007 |

Abbreviations: CI, confidence interval; S.E., standard error of the mean

Results are expressed as standardized IQ-scores (M=100, SD=15). Higher numbers indicate better performance. Estimated marginal means are presented.

Raw P values are presented. The Q value represents the false discovery rate adjusted P value.

## 1.18 Full Scale IQ in relation to the estimated fetal dose of radiation (expressed in milligrays) for 14 children exposed to radiotherapy during pregnancy (eFigure 4)


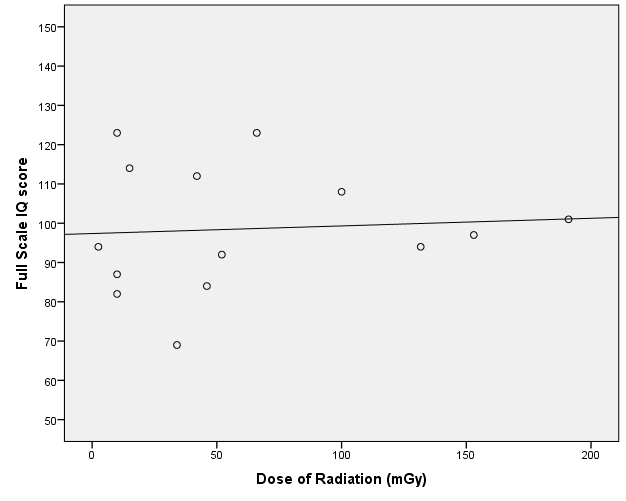


The dose program “Peridose” developed by van der Giessen was used to estimate the fetal radiation dose.[1]

Full Scale IQ was not related to the estimated fetal dose of radiation (r=0.19, P=0.52).

## 1.19 Verbal IQ in children exposed to anthracyclines, FAC/FEC chemotherapy, taxanes or platinum-based treatments compared to matched controls using ANCOVA with parental education levels as covariates (eTable 15)

| Measurement | No. | Children exposed to anthracyclines (N=75)* | | | | Control group (N=75) | | | |  |
| --- | --- | --- | --- | --- | --- | --- | --- | --- | --- | --- |
|  |  | **Mean** | S.E. | 95% CI | | **Mean** | S.E. | 95% CI | | Between-group difference of the means |
|  |  |  |  | Lower | Upper |  |  | Lower | Upper |  |
| Verbal IQ | 132 | **101.1** | 3.8 | 93.6 | 108.6 | **107.8** | 4.0 | 99.9 | 115.7 | -6.7 |

| Measurement | No. | Children exposed to (F)AC or (F)E(C) (N=60)* | | | | Control group (N=60) | | | |  |
| --- | --- | --- | --- | --- | --- | --- | --- | --- | --- | --- |
|  |  | **Mean** | S.E. | 95% CI | | **Mean** | S.E. | 95% CI | | Between-group difference of the means |
|  |  |  |  | Lower | Upper |  |  | Lower | Upper |  |
| Verbal IQ | 108 | **101.5** | 3.4 | 94.8 | 108.3 | **107.3** | 3.7 | 100.1 | 114.6 | -5.8 |

| Measurement | No. | Children exposed to taxanes (N=17)* | | | | Control group (N=17) | | | |  |
| --- | --- | --- | --- | --- | --- | --- | --- | --- | --- | --- |
|  |  | **Mean** | S.E. | 95% CI | | **Mean** | S.E. | 95% CI | | Between-group difference of the means |
|  |  |  |  | Lower | Upper |  |  | Lower | Upper |  |
| Verbal IQ | 33 | **104.2** | 6.0 | 91.8 | 116.6 | **113.4** | 5.1 | 103.0 | 123.9 | -9.2 |

| Measurement | No. | Children exposed to platin-based treatments (N=20)* | | | | Control group (N=20) | | | |  |
| --- | --- | --- | --- | --- | --- | --- | --- | --- | --- | --- |
|  |  | **Mean** | S.E. | 95% CI | | **Mean** | S.E. | 95% CI | | Between-group difference of the means |
|  |  |  |  | Lower | Upper |  |  | Lower | Upper |  |
| Verbal IQ | 35 | **105.0** | 4.3 | 96.2 | 113.8 | **113.8** | 5.2 | 103.2 | 124.4 | -8.8 |

Abbreviations: CI, confidence interval; (F)AC, 5-fluorouracil, doxorubicin, cyclophosphamide; (F)E(C), 5-fluorouracil, epirubicin, cyclophosphamide; S.E., standard error of the mean

Results are expressed as standardized IQ-scores (M=100, SD=15). Higher numbers indicate better performance. Estimated marginal means are presented.

*Some children had prenatal exposure to a combination of treatment options (e.g., anthracyclines followed by taxanes or taxanes plus platin-based treatment) and therefore are included in more than one group.

We cannot directly compare the results of children exposed to anthracyclines with those of children exposed to taxanes or to platin-based treatments, as the groups may differ with respect to the distribution of gender, gestational age, test age, country and language and some children are included in more than one group. The results of one group should be compared to their matched controls. Although the groups of children exposed to taxanes and platin-based treatments are small, we can have a look at the size of the between-group differences for the different types of chemotherapy to see whether the between-group differences are larger for one type of chemotherapy compared to the others. We can conclude from these tables that the size of the between-group differences seems to be comparable for the different types of chemotherapy (given the small sample sizes), so it does not seem that one type of chemotherapy has a larger effect on Verbal IQ than the others.

We compared the results of children exposed to (F)AC / (F)E(C) chemotherapy to those of their matched controls, because these agents are used in the treatment of breast cancer, which constitutes the largest group of patients in our study.

## 1.20 Verbal IQ in study children whose mother died and those with surviving mothers compared to matched controls using ANCOVA with parental education levels as covariates (eTable 16)

| Measurement | No. | Children whose mother died (N=26) | | | | Control group (N=26) | | | |  |
| --- | --- | --- | --- | --- | --- | --- | --- | --- | --- | --- |
|  |  | **Mean** | S.E. | 95% CI | | **Mean** | S.E. | 95% CI | | Between-group difference of the means |
|  |  |  |  | Lower | Upper |  |  | Lower | Upper |  |
| Verbal IQ | 45 | **85.7** | 4.2 | 77.2 | 94.1 | **100.8** | 4.4 | 91.8 | 109.8 | -15.1 |

| Measurement | No. | Children with surviving mothers (N=106) | | | | Control group (N=106) | | | |  |
| --- | --- | --- | --- | --- | --- | --- | --- | --- | --- | --- |
|  |  | **Mean** | S.E. | 95% CI | | **Mean** | S.E. | 95% CI | | Between-group difference of the means |
|  |  |  |  | Lower | Upper |  |  | Lower | Upper |  |
| Verbal IQ | 191 | **101.2** | 2.1 | 97.1 | 105.2 | **106.1** | 2.2 | 101.7 | 110.5 | -4.9 |

Abbreviations: CI, confidence interval; (F)AC, 5-fluorouracil, doxorubicin, cyclophosphamide; S.E., standard error of the mean

Results are expressed as standardized IQ-scores (M=100, SD=15). Higher numbers indicate better performance. Estimated marginal means are presented.

We cannot directly compare the results of children whose mother died and those with surviving mothers, as the groups may differ with respect to the distribution of gender, gestational age, test age, country and language. The results of one group should be compared to their matched controls. Although the group of children whose mother died is small, we can have a look at the size of the between-group differences for children whose mother died and those with surviving mothers and see whether the between-group differences are larger for one of the groups. We can conclude from these tables that the size of the between-group difference is larger with respect to Verbal IQ for children whose mother died than for children with surviving mothers compared to their matched controls. It thus seems that death of the mother may adversely impact on Verbal IQ. However, caution is indicated given the small sample size of children whose mother died.

## 1.21 Memory outcomes in children from the cancer in pregnancy group compared to matched controls using ANCOVA with parental education levels as covariates (eTable 17)

| CMS subtask | Measurement | Minimum to maximum raw score | No. | Cancer in pregnancy group (N=132) | | | | Control group (N=132) | | | | Type 3 test of fixed effects | | | |
| --- | --- | --- | --- | --- | --- | --- | --- | --- | --- | --- | --- | --- | --- | --- | --- |
|  |  |  |  | **Mean** | S.E. | 95% CI | | **Mean** | S.E. | 95% CI | | F | P value | Q value | Partial eta squared |
|  |  |  |  |  |  | Lower | Upper |  |  | Lower | Upper |  |  |  |  |
| Numbers forward | Verbal memory span | 0-16 | 238 | **5.06** | 0.22 | 4.64 | 5.48 | **5.47** | 0.24 | 5.01 | 5.94 | 3.90 | 0.05 | 0.17 | 0.017 |
| Numbers backward | Verbal working memory | 0-14 | 238 | **2.65** | 0.16 | 2.34 | 2.95 | **2.71** | 0.17 | 2.38 | 3.05 | 0.19 | 0.67 | 0.71 | 0.001 |
| Picture Locations | Visuospatial memory span | 0-30 | 238 | **24.26** | 0.39 | 23.49 | 25.03 | **24.93** | 0.43 | 24.08 | 25.77 | 3.04 | 0.08 | 0.21 | 0.013 |
| Dot Locations | Visuospatial short-term memory | 0-6 | 237 | **4.33** | 0.19 | 3.96 | 4.70 | **4.69** | 0.21 | 4.28 | 5.10 | 3.74 | 0.05 | 0.17 | 0.016 |
| Dot Locations | Visuospatial long-term memory | 0-6 | 241 | **3.95** | 0.20 | 3.56 | 4.34 | **4.50** | 0.22 | 4.07 | 4.93 | 8.09 | 0.005 | 0.045 | 0.034 |
| Faces | Short-term memory for faces | 0-36 | 240 | **25.29** | 0.51 | 24.29 | 26.28 | **24.80** | 0.56 | 23.70 | 25.90 | 1.00 | 0.32 | 0.74 | 0.004 |
| Faces | Long-term memory for faces | 0-36 | 238 | **24.69** | 0.54 | 23.63 | 25.75 | **24.82** | 0.60 | 23.65 | 26.00 | 0.06 | 0.80 | 0.92 | 0.000 |

Abbreviations: CI, confidence interval; S.E., standard error of the mean

Results are expressed as raw subtest scores. Higher numbers indicate better performance. Estimated marginal means are presented.

Raw P values are presented. The Q value represents the false discovery rate adjusted P value.

## 1.22 Memory outcomes in chemotherapy-exposed children compared to matched controls using ANCOVA with parental education levels as covariates (eTable 18)

| CMS subtask | Measurement | Minimum to maximum raw score | No. | Chemotherapy group (N=97) | | | | Control group (N=97) | | | | Type 3 test of fixed effects | | | |
| --- | --- | --- | --- | --- | --- | --- | --- | --- | --- | --- | --- | --- | --- | --- | --- |
|  |  |  |  | **Mean** | S.E. | 95% CI | | **Mean** | S.E. | 95% CI | | F | P value | Q value | Partial eta squared |
|  |  |  |  |  |  | Lower | Upper |  |  | Lower | Upper |  |  |  |  |
| Numbers forward | Verbal memory span | 0-16 | 172 | **5.09** | 0.41 | 4.28 | 5.90 | **5.43** | 0.43 | 4.59 | 6.27 | 1.96 | 0.16 | 0.48 | 0.012 |
| Numbers backward | Verbal working memory | 0-14 | 172 | **2.90** | 0.30 | 2.30 | 3.50 | **2.94** | 0.32 | 2.32 | 3.56 | 0.06 | 0.80 | 0.85 | 0.000 |
| Picture Locations | Visuospatial memory span | 0-30 | 172 | **24.67** | 0.74 | 23.22 | 26.13 | **25.37** | 0.77 | 23.86 | 26.89 | 2.60 | 0.11 | 0.39 | 0.016 |
| Dot Locations | Visuospatial short-term memory | 0-6 | 171 | **4.62** | 0.37 | 3.90 | 5.35 | **5.18** | 0.38 | 4.42 | 5.93 | 6.40 | 0.01 | 0.07 | 0.038 |
| Dot Locations | Visuospatial long-term memory | 0-6 | 175 | **4.04** | 0.38 | 3.28 | 4.80 | **4.68** | 0.40 | 3.89 | 5.47 | 7.99 | 0.005 | 0.045 | 0.046 |
| Faces | Short-term memory for faces | 0-36 | 175 | **26.37** | 1.00 | 24.39 | 28.35 | **26.65** | 0.96 | 24.75 | 28.55 | 0.25 | 0.62 | 0.95 | 0.001 |
| Faces | Long-term memory for faces | 0-36 | 174 | **26.78** | 1.08 | 24.66 | 28.91 | **25.77** | 1.03 | 23.73 | 27.81 | 2.77 | 0.10 | 0.69 | 0.016 |

Abbreviations: CI, confidence interval; S.E., standard error of the mean

Results are expressed as raw subtest scores. Higher numbers indicate better performance. Estimated marginal means are presented.

Raw P values are presented. The Q value represents the false discovery rate adjusted P value.

## 1.23 Attention outcomes in children from the cancer in pregnancy group compared to matched controls using ANCOVA with parental education levels as covariates (eTable 19)

| ANT subtask | Measurement | No. | Cancer in pregnancy group (N=132) | | | | Control group (N=132) | | | | Type 3 test of fixed effects | | | |  |
| --- | --- | --- | --- | --- | --- | --- | --- | --- | --- | --- | --- | --- | --- | --- | --- |
|  |  |  | **Mean** | S.E. | 95% CI | | **Mean** | S.E. | 95% CI | | F | P value | Q value | Partial eta squared |  |
|  |  |  |  |  | Lower | Upper |  |  | Lower | Upper |  |  |  |  |  |
| Baseline Speed | **Alertness** | | | | | | | | | | | | | | |
|  | Mean RT of dominant and non-dominant hand (ms) | 237 | **545.8** | 18.6 | 509.1 | 582.5 | **514.3** | 20.1 | 474.7 | 553.9 | 3.62 | 0.06 | 0.17 | 0.016 |  |
| Go-NoGo | **Response inhibition** | | | | | | | | | | | | | | |
|  | RT hits (ms) | 236 | **685.5** | 16.4 | 653.2 | 717.7 | **670.3** | 17.6 | 635.7 | 704.9 | 1.10 | 0.30 | 0.46 | 0.005 |  |
|  | Number of false alarms (%) | 236 | **14.6** | 1.8 | 10.9 | 18.2 | **12.3** | 2.0 | 8.4 | 16.2 | 1.87 | 0.17 | 0.37 | 0.008 |  |
| Memory Search Objects 2 keys | **Divided attention** | | | | | | | | | | | | | | |
|  | Total RT (ms) | 230 | **6034.4** | 187.9 | 5664.1 | 6404.6 | **6051.9** | 201.5 | 5654.7 | 6449.0 | 0.01 | 0.92 | 0.92 | 0.000 |  |
|  | Total number of errors (%) | 230 | **7.6** | 0.7 | 6.2 | 9.0 | **7.9** | 0.8 | 6.4 | 9.4 | 0.22 | 0.64 | 0.71 | 0.001 |  |
|  | Effect of memory load on RT (ms) | 230 | **445.1** | 42.9 | 360.5 | 529.7 | **400.5** | 46.0 | 309.8 | 491.3 | 1.36 | 0.25 | 0.42 | 0.006 |  |
|  | Effect of memory load on accuracy | 230 | **0.32** | 0.97 | -1.59 | 2.24 | **-0.51** | 1.04 | -2.57 | 1.54 | 0.93 | 0.34 | 0.50 | 0.004 |  |
| Focused Attention Objects 2 keys | **Selective attention** | | | | | | | | | | | | | | |
|  | Total RT (ms) | 231 | **4867.5** | 173.6 | 4525.4 | 5209.7 | **4917.8** | 187.0 | 4549.2 | 5286.3 | 0.11 | 0.75 | 0.92 | 0.000 |  |
|  | Total number of errors (%) | 233 | **7.4** | 1.1 | 5.3 | 9.6 | **6.9** | 1.2 | 4.6 | 9.3 | 0.27 | 0.61 | 0.74 | 0.001 |  |
|  | Effect of distraction on RT (ms) | 233 | **-0.3** | 43.6 | -86.2 | 85.7 | **8.1** | 46.9 | -84.3 | 100.6 | 0.05 | 0.83 | 0.83 | 0.000 |  |
|  | Effect of distraction on accuracy | 233 | **1.40** | 0.71 | 0.00 | 2.80 | **1.70** | 0.76 | 0.19 | 3.20 | 0.22 | 0.64 | 0.71 | 0.001 |  |

Abbreviations: CI, confidence interval; CR, correct rejections; FA, false alarms; P, percentage; RT, reaction time; S.E., standard error of the mean.

Results are expressed as raw subtest scores. Reaction times are expressed in milliseconds (ms), while numbers of errors are expressed in percentages. Higher numbers indicate worse performance. Estimated marginal means are presented. Raw P values are presented. The Q value represents the false discovery rate adjusted P value.

The effect of memory load on reaction time is calculated as ((RT hits + RT CR)_part2_ – (RT hits + RT CR)_part1_)/2. Higher numbers indicate a larger effect of memory load on reaction time. The effect of memory load on accuracy is calculated as ((P-MI + P-FA)_part2_ – (P-MI + P-FA)_part1_)/2. Higher numbers indicate a larger effect of memory load on accuracy. The effect of distraction on reaction time is calculated as RT CR [irrelevant target] – RT CR [non-target]. Higher numbers indicate a larger effect of distraction on reaction time. The effect of distraction on accuracy is calculated as P-FA[irrelevant target] – P-FA[non-target]. Higher numbers indicate a larger effect of distraction on accuracy.

## 1.24 Attention outcomes in chemotherapy-exposed children compared to matched controls using ANCOVA with parental education levels as covariates (eTable 20)

| ANT subtask | Measurement | No. | Chemotherapy group (N=97) | | | | Control group (N=97) | | | | Type 3 test of fixed effects | | | |  |
| --- | --- | --- | --- | --- | --- | --- | --- | --- | --- | --- | --- | --- | --- | --- | --- |
|  |  |  | **Mean** | S.E. | 95% CI | | **Mean** | S.E. | 95% CI | | F | P value | Q value | Partial eta squared |  |
|  |  |  |  |  | Lower | Upper |  |  | Lower | Upper |  |  |  |  |  |
| Baseline Speed | **Alertness** | | | | | | | | | | | | | | |
|  | Mean RT of dominant and non-dominant hand (ms) | 173 | **522.2** | 31.2 | 460.7 | 583.8 | **500.4** | 32.4 | 436.5 | 564.4 | 1.41 | 0.24 | 0.49 | 0.008 |  |
| Go-NoGo | **Response inhibition** | | | | | | | | | | | | | | |
|  | RT hits (ms) | 173 | **688.5** | 29.5 | 630.3 | 746.8 | **693.2** | 30.7 | 632.6 | 753.7 | 0.07 | 0.79 | 0.95 | 0.000 |  |
|  | Number of false alarms (%) | 173 | **15.2** | 3.2 | 8.9 | 21.6 | **13.0** | 3.3 | 6.4 | 19.6 | 1.39 | 0.24 | 0.48 | 0.008 |  |
| Memory Search Objects 2 keys | **Divided attention** | | | | | | | | | | | | | | |
|  | Total RT (ms) | 167 | **6138.7** | 342.6 | 5462.2 | 6815.2 | **6095.6** | 355.9 | 5392.7 | 6798.5 | 0.04 | 0.83 | 0.95 | 0.000 |  |
|  | Total number of errors (%) | 167 | **9.3** | 1.3 | 6.7 | 11.9 | **9.9** | 1.4 | 7.2 | 12.6 | 0.59 | 0.44 | 0.57 | 0.003 |  |
|  | Effect of memory load on RT (ms) | 167 | **417.6** | 74.7 | 270.0 | 565.2 | **358.2** | 77.7 | 204.9 | 511.6 | 1.75 | 0.19 | 0.48 | 0.011 |  |
|  | Effect of memory load on accuracy | 167 | **-1.02** | 1.68 | -4.35 | 2.30 | **-1.89** | 1.75 | -5.34 | 1.57 | 0.73 | 0.40 | 0.55 | 0.005 |  |
| Focused Attention Objects 2 keys | **Selective attention** | | | | | | | | | | | | | | |
|  | Total RT (ms) | 169 | **4803.4** | 310.1 | 4191.1 | 5415.7 | **4792.2** | 322.7 | 4155.0 | 5429.4 | 0.00 | 0.95 | 0.95 | 0.000 |  |
|  | Total number of errors (%) | 170 | **7.4** | 1.9 | 3.7 | 11.1 | **6.5** | 2.0 | 2.6 | 10.4 | 0.61 | 0.43 | 0.95 | 0.004 |  |
|  | Effect of distraction on RT (ms) | 170 | **26.3** | 79.0 | -129.7 | 182.3 | **20.5** | 82.2 | -141.9 | 182.9 | 0.02 | 0.90 | 0.90 | 0.000 |  |
|  | Effect of distraction on accuracy | 170 | **0.70** | 0.89 | -1.06 | 2.45 | **1.28** | 0.92 | -0.54 | 3.10 | 1.24 | 0.27 | 0.48 | 0.008 |  |

Abbreviations: CI, confidence interval; CR, correct rejections; FA, false alarms; P, percentage; RT, reaction time; S.E., standard error of the mean.

Results are expressed as raw subtest scores. Reaction times are expressed in milliseconds (ms), while numbers of errors are expressed in percentages. Higher numbers indicate worse performance. Estimated marginal means are presented. Raw P values are presented. The Q value represents the false discovery rate adjusted P value.

The effect of memory load on reaction time is calculated as ((RT hits + RT CR)_part2_ – (RT hits + RT CR)_part1_)/2. Higher numbers indicate a larger effect of memory load on reaction time. The effect of memory load on accuracy is calculated as ((P-MI + P-FA)_part2_ – (P-MI + P-FA)_part1_)/2. Higher numbers indicate a larger effect of memory load on accuracy. The effect of distraction on reaction time is calculated as RT CR [irrelevant target] – RT CR [non-target]. Higher numbers indicate a larger effect of distraction on reaction time. The effect of distraction on accuracy is calculated as P-FA[irrelevant target] – P-FA[non-target]. Higher numbers indicate a larger effect of distraction on accuracy.

## 1.25 Behavior problems in children from the cancer in pregnancy group compared to matched controls using ANCOVA with parental education levels as covariates (eTable 21)

| Measurement | No. | Cancer in pregnancy group (N=132) | | | | Control group (N=132) | | | | Type 3 test of fixed effects | | | |
| --- | --- | --- | --- | --- | --- | --- | --- | --- | --- | --- | --- | --- | --- |
|  |  | **Mean** | S.E. | 95% CI | | **Mean** | S.E. | 95% CI | | F | P value | Q value | Partial eta squared |
|  |  |  |  | Lower | Upper |  |  | Lower | Upper |  |  |  |  |
| Internalizing problems | 230 | **50.8** | 1.3 | 48.2 | 53.4 | **51.7** | 1.5 | 48.8 | 54.6 | 0.51 | 0.48 | 0.66 | 0.002 |
| Externalizing problems | 230 | **52.0** | 1.3 | 49.4 | 54.6 | **53.7** | 1.4 | 51.0 | 56.6 | 1.79 | 0.18 | 0.37 | 0.008 |
| Total problems | 230 | **51.3** | 1.3 | 48.7 | 54.0 | **52.9** | 1.5 | 49.9 | 55.8 | 1.41 | 0.24 | 0.74 | 0.006 |

Abbreviations: CI, confidence interval; S.E., standard error of the mean

Results are expressed as standardized T-scores (M=50, SD=15). Higher numbers indicate more behavior problems. Estimated marginal means are presented.

Raw P values are presented. The Q value represents the false discovery rate adjusted P value.

## 1.26 Behavior problems in chemotherapy-exposed children compared to matched controls using ANCOVA with parental education levels as covariates (eTable 22)

| Measurement | No. | Chemotherapy group (N=97) | | | | Control group (N=97) | | | | Type 3 test of fixed effects | | | |
| --- | --- | --- | --- | --- | --- | --- | --- | --- | --- | --- | --- | --- | --- |
|  |  | **Mean** | S.E. | 95% CI | | **Mean** | S.E. | 95% CI | | F | P value | Q value | Partial eta squared |
|  |  |  |  | Lower | Upper |  |  | Lower | Upper |  |  |  |  |
| Internalizing problems | 164 | **52.0** | 2.7 | 46.6 | 57.3 | **51.1** | 2.8 | 45.4 | 56.7 | 0.33 | 0.57 | 0.64 | 0.002 |
| Externalizing problems | 164 | **53.7** | 2.6 | 58.7 | 58.8 | **55.3** | 2.7 | 50.0 | 60.6 | 1.02 | 0.31 | 0.49 | 0.006 |
| Total problems | 164 | **52.7** | 2.7 | 47.3 | 58.0 | **53.4** | 2.8 | 47.9 | 59.0 | 0.22 | 0.64 | 0.95 | 0.001 |

Abbreviations: CI, confidence interval; S.E., standard error of the mean

Results are expressed as standardized T-scores (M=50, SD=15). Higher number indicate more behavior problems. Estimated marginal means are presented.

Raw P values are presented. The Q value represents the false discovery rate adjusted P value.

## 1.27 Behavior problems in study children whose mother died and those with surviving mothers compared to matched controls using ANCOVA with parental education levels as covariates (eTable 23)

| Measurement | No. | Children whose mother died (N=26) | | | | Control group (N=26) | | | |  |
| --- | --- | --- | --- | --- | --- | --- | --- | --- | --- | --- |
|  |  | **Mean** | S.E. | 95% CI | | **Mean** | S.E. | 95% CI | | Between-group difference of the means |
|  |  |  |  | Lower | Upper |  |  | Lower | Upper |  |
| Internalizing problems | 47 | **48.5** | 2.8 | 42.9 | 54.2 | **54.4** | 3.2 | 47.9 | 60.8 | -5.9 |
| Externalizing problems | 47 | **49.5** | 3.0 | 43.3 | 55.6 | **53.9** | 3.4 | 46.9 | 60.8 | -4.4 |

| Measurement | No. | Children with surviving mothers (N=106) | | | | Control group (N=106) | | | |  |
| --- | --- | --- | --- | --- | --- | --- | --- | --- | --- | --- |
|  |  | **Mean** | S.E. | 95% CI | | **Mean** | S.E. | 95% CI | | Between-group difference of the means |
|  |  |  |  | Lower | Upper |  |  | Lower | Upper |  |
| Internalizing problems | 183 | **50.9** | 1.5 | 47.9 | 53.9 | **50.7** | 1.7 | 47.4 | 54.0 | 0.2 |
| Externalizing problems | 183 | **52.8** | 1.5 | 49.8 | 55.7 | **53.7** | 1.6 | 50.5 | 57.0 | -0.9 |

Abbreviations: CI, confidence interval; S.E., standard error of the mean

Results are expressed as standardized T-scores (M=50, SD=15). Higher numbers indicate more behavior problems. Estimated marginal means are presented.

We cannot directly compare the results of children whose mother died and those with surviving mothers, as the groups may differ with respect to the distribution of gender, gestational age, test age, country and language. The results of one group should be compared to their matched controls. Although the group of children whose mother died is small, we can have a look at the size of the between-group differences for children whose mother died and those with surviving mothers and see whether the between-group differences are larger for one of the groups. We can conclude from these tables that the size of the between-group difference is somewhat larger with respect to internalizing and externalizing problems for children whose mother died than for children with surviving mothers compared to their matched controls, although none of the groups scored significantly higher or lower than their matched controls. Caution is indicated given the small sample size of children whose mother died.

## 1.28 Echocardiographic measurements, pulsed tissue Doppler imaging (TDI) and speckle-tracking measurements in children exposed to anthracyclines compared to matched controls using ANOVA (eTable 24)

| Measurement | No. | Anthracycline-exposed group (N=59) | | | | | Control group (N=59) | | | | Type 3 test of fixed effects | | | |
| --- | --- | --- | --- | --- | --- | --- | --- | --- | --- | --- | --- | --- | --- | --- |
|  |  | **Mean** | | S.E. | 95% CI | | **Mean** | S.E. | 95% CI | | F | P value | Q value | Partial eta squared |
|  |  |  |  |  | Lower | Upper |  |  | Lower | Upper |  |  |  |  |
| Body-surface area (m²) | 116 | **0.85** | | 0.01 | 0.83 | 0.87 | **0.84** | 0.01 | 0.82 | 0.86 | 0.43 | 0.51 | 0.79 | 0.004 |
| Blood pressure (mm Hg) |  | |  | | | | | | | | | | | |
| Systolic | 104 | **103.67** | | 1.40 | 100.89 | 106.46 | **100.12** | 1.25 | 97.64 | 102.60 | 3.58 | 0.06 | 0.34 | 0.034 |
| Diastolic | 104 | **61.83** | | 1.28 | 59.30 | 64.36 | **55.85** | 1.14 | 53.59 | 58.10 | 12.20 | <0.001 | 0.02 | 0.107 |
| Heart rate (beats/min) | 118 | **82.68** | | 1.54 | 79.64 | 85.72 | **80.88** | 1.54 | 77.84 | 83.92 | 0.68 | 0.41 | 0.77 | 0.006 |
| Left ventricular shortening fraction (%) | 118 | **36.32** | | 0.57 | 35.20 | 37.44 | **37.05** | 0.57 | 35.93 | 38.17 | 0.84 | 0.36 | 0.77 | 0.007 |
| Left ventricular ejection fraction (%) | 118 | **66.78** | | 0.72 | 65.36 | 68.20 | **67.76** | 0.72 | 66.35 | 69.18 | 0.94 | 0.33 | 0.77 | 0.008 |
| End-diastolic diameter (cm) |  | |  | | | | | | | | | | | |
| Left ventricular | 118 | **3.59** | | 0.04 | 3.52 | 3.66 | **3.65** | 0.04 | 3.58 | 3.72 | 1.08 | 0.30 | 0.77 | 0.009 |
| Right ventricular | 117 | **1.57** | | 0.03 | 1.52 | 1.63 | **1.60** | 0.03 | 1.55 | 1.66 | 0.66 | 0.42 | 0.77 | 0.006 |
| Left ventricular posterior-wall thickness (cm) | 118 | **0.53** | | 0.01 | 0.51 | 0.55 | **0.54** | 0.01 | 0.52 | 0.56 | 0.11 | 0.74 | 0.97 | 0.001 |
| Interventricular septum thickness (cm) | 118 | **0.53** | | 0.01 | 0.51 | 0.55 | **0.54** | 0.01 | 0.52 | 0.56 | 0.58 | 0.45 | 0.77 | 0.005 |
| Mitral valve E velocity (m/s) | 116 | **1.02** | | 0.02 | 0.98 | 1.06 | **1.08** | 0.02 | 1.04 | 1.12 | 5.39 | 0.02 | 0.26 | 0.045 |
| Mitral valve A velocity (m/s) | 115 | **0.53** | | 0.01 | 0.50 | 0.55 | **0.57** | 0.01 | 0.54 | 0.59 | 4.10 | 0.05 | 0.34 | 0.035 |
| Mitral valve E/A ratio | 115 | **2.01** | | 0.06 | 1.89 | 2.12 | **1.98** | 0.06 | 1.87 | 2.09 | 0.12 | 0.73 | 0.97 | 0.001 |
| Basal segment of left ventricular lateral wall (cm/sec)* |  | |  | | | | | | | | | | | |
| Peak systolic velocity | 105† | **8.97** | | 0.28 | 8.40 | 9.53 | **8.90** | 0.26 | 8.39 | 9.42 | 0.03 | 0.87 | 0.97 | 0.000 |
| Peak early diastolic velocity | 106† | **17.55** | | 0.38 | 16.80 | 18.29 | **17.51** | 0.35 | 16.82 | 18.20 | 0.01 | 0.95 | 0.97 | 0.000 |
| Peak late diastolic velocity | 106† | **6.01** | | 0.25 | 5.52 | 6.49 | **5.79** | 0.24 | 5.33 | 6.26 | 0.40 | 0.53 | 0.79 | 0.004 |
| Basal segment of interventricular septum (cm/sec)* |  | |  | | | | | | | | | | | |
| Peak systolic velocity | 110† | **7.26** | | 0.18 | 6.89 | 7.62 | **7.70** | 0.17 | 7.36 | 8.03 | 3.01 | 0.09 | 0.34 | 0.027 |
| Peak early diastolic velocity | 110† | **13.90** | | 0.24 | 13.42 | 14.38 | **13.54** | 0.23 | 13.09 | 13.99 | 1.17 | 0.28 | 0.77 | 0.011 |
| Peak late diastolic velocity | 109† | **5.64** | | 0.16 | 5.33 | 5.95 | **5.65** | 0.14 | 5.37 | 5.93 | 0.002 | 0.97 | 0.97 | 0.000 |
| Basal segment of right ventricular lateral wall (cm/sec)* |  | |  | | | | | | | | | | | |
| Peak systolic velocity | 105† | **12.82** | | 0.28 | 12.28 | 13.37 | **12.87** | 0.26 | 12.35 | 13.38 | 0.01 | 0.91 | 0.97 | 0.000 |
| Peak early diastolic velocity | 106† | **15.78** | | 0.38 | 15.03 | 16.53 | **15.75** | 0.36 | 15.05 | 16.46 | 0.002 | 0.96 | 0.97 | 0.000 |
| Peak late diastolic velocity | 105† | **9.25** | | 0.31 | 8.64 | 9.85 | **9.36** | 0.29 | 8.78 | 9.93 | 0.07 | 0.80 | 0.97 | 0.001 |
| Global left ventricular longitudinal strain (%) | 98† | **20.60** | | 0.34 | 19.92 | 21.27 | **21.00** | 0.33 | 20.34 | 21.66 | 0.71 | 0.40 | 0.77 | 0.007 |
| Global left ventricular circumferential strain (%) | 86† | **21.69** | | 0.55 | 20.59 | 22.78 | **20.40** | 0.45 | 19.51 | 21.29 | 3.27 | 0.07 | 0.34 | 0.037 |

| Abbreviations: CI, confidence interval; S.E., standard error of the mean  Raw P values are presented. The Q value represents the false discovery rate adjusted P value. |
| --- |

*Measurements were obtained with the use of tissue Doppler imaging.

†Data were not included when tracking could not be performed owing to poor image quality.

## 1.29 General health problems of study and control children, registered by the parents (eTable 25)

|  |  | **Cancer in pregnancy group** | | **Control group** | |
| --- | --- | --- | --- | --- | --- |
|  |  | N | % | N | % |
| Medical | Respiratory disorders (asthma, infections) | 20 | 16.3 | 19 | 16.0 |
|  | Recurrent otitis | 13 | 10.6 | 16 | 13.4 |
|  | Skin disorders (eczema, café au lait, hemangioma, warts, impetigo) | 14 | 11.4 | 16 | 13.4 |
|  | Allergy | 15 | 12.2 | 16 | 13.4 |
|  | Urinary tract disorders (dilatation of kidney, pyelonephritis, infections e.g. cystitis, reflux) | 8 | 6.5 | 7 | 5.9 |
|  | Heart and vessel disorders (Wolff-Parkinson-White, factor V Leiden) | 2 | 1.6 | 1 | 0.8 |
|  | Gastro-intestinal disorders (gastro-enteritis, diarrhea, reflux, constipation, coeliac disease, lactose intolerance) | 6 | 4.9 | 7 | 5.9 |
|  | Genital tract disorders (phimosis, fungal infections, cryptorchidism) | 5 | 4.1 | 4 | 3.4 |
|  | Hormonal disorders | 0 | 0.0 | 0 | 0.0 |
|  | Neurological disorders (epilepsia, feverish convulsions, frequent headaches) | 3 | 2.4 | 2 | 1.7 |
|  | Wearing glasses at the age of 6 years* | 17 | 14.9 | 6 | 5.0 |
| Surgical | Ear tube surgery | 12 | 9.8 | 10 | 8.4 |
|  | Tonsillectomy and/or adenoidectomy and/or polypectomy | 17 | 13.8 | 10 | 8.4 |
|  | Orchidopexy | 3 | 2.4 | 0 | 0.0 |
|  | Circumcision / broadening of the foreskin for phimosis or adhesion of the foreskin | 1 | 0.8 | 2 | 1.7 |
|  | Tear duct drainage | 1 | 0.8 | 0 | 0.0 |
|  | Inguinal hernia closure | 3 | 2.4 | 3 | 2.5 |
|  | Umbilical hernia closure | 1 | 0.8 | 0 | 0.0 |
|  | Strabismus | 2 | 1.6 | 1 | 0.8 |
|  | Dental surgery | 2 | 1.6 | 4 | 3.4 |
|  | Pneumothorax | 1 | 0.8 | 1 | 0.8 |
|  | Other (study group: nose correction for fracture of nasal bone, urethrocystoscopy, for dilatation of the kidney, for lazy eye, for hymen imperforatus, removal of knot between kidney and urethra, frenulotomy to correct tongue-tie, for anorectal atresy, removal of accessory auricle; control group: gastroscopy with biopsy, for hypospadias, ear correction, trigger finger, congenital club foot, hordeolum on inside of eyelid) | 9 | 7.3 | 7 | 5.9 |
| Paramedical care | Physiotherapy / Osteopathy | 4 | 3.3 | 9 | 7.6 |
|  | Speech therapy | 12 | 9.8 | 5 | 4.2 |
|  | Psychotherapy | 2 | 1.6 | 0 | 0.0 |

General health questionnaires were available for 123/132 study children and for 119/132 control children.

*Data on the need for glasses was available for 114/132 study children and for 118/132 control children.

## 1.30 Hearing loss in children prenatally exposed to cisplatin (eTable 26)

| Patient number | Treatment characteristics | Gestational age at exposure (weeks) | Number of cycles during pregnancy | Total dose of cisplatin in pregnancy | Audiometry results |
| --- | --- | --- | --- | --- | --- |
| 1 | Cisplatin (70mg/m²) + paclitaxel (90 mg/m²) | 26.4-31.4 | 6 | 420mg/m² | Bilateral hearing loss diagnosed at birth. Case described by Geijteman et al.[2] |
| 2 | Cisplatin (75mg/m²) + paclitaxel (175mg/m²) | 16.9-32.0 | 6 | 450mg/m² | Bilateral hearing loss in low and high regions (up to 50dB) at the age of 6 years |
| 3 | Cisplatin (100mg/m²) | 27.4-33.6 | 3 | 300mg/m² | Bilateral hearing loss in the high regions (up to 100 dB) at the age of 6 years. Case described by Amant et al.[3] |
| 4 | Cisplatin (75mg/m²) | 17.3-27.3 | 6 | 450mg/m² | Normal results at the age of 6 years |
| 5 (twin) | Cisplatin (20mg/m²) | 20.0-26.0 | 3 | 60mg/m² | Normal results at the age of 5 years in both children |
| 6 | Cisplatin (40mg/m²) | 16.9-22.9 | 6 | 240mg/m² | Normal results at the age of 6 years |
| 7 | Cisplatin (50mg/m²) + 5-FU (200mg/m²) | 30.3-33.4 | 2 | 100mg/m² | Normal results at the age of 6 years |
| 8 | Cisplatin (75mg/m²) | 16.9-32.1 | 6 | 450mg/m² | No data available |
| 9 | Cisplatin (dose unknown) | 23.3-32.0 | 4 | Unknown | No data available |
| 10 | Cisplatin (dose unknown) | 20.0-35.3 | 6 | Unknown | No data available |
| 11 | Cisplatin (dose unknown) | 27.0-33.0 | 3 | Unknown | No data available |
| 12 | Cisplatin (30mg/m²) + epirubicin (30mg/m²) | 20.6-27.6 | 8 | 240mg/m² | No data available |
| 13 | Cisplatin (70mg/m²) + paclitaxel (70mg/m²) | 20.0-25.0 (cisplatin + paclitaxel) and 29.0-30.0 (paclitaxel) | 6 cisplatin and paclitaxel + 2 paclitaxel only | 420mg/m² | No data available |

## Abbreviations: 5FU, 5-fluorouracil

## 1.31 Abnormalities observed during history taking and general pediatric and clinical neurological examination in 103 study children undergoing examination (eTable 27)

| **Treatment during pregnancy** | **Abnormality observed (N=103)*** |
| --- | --- |
| Chemotherapy | - Epilepsia from 11 months until 4 years with feverish convulsions (N=1) - Autism spectrum disorder with delayed language development, diagnosed at the age of 4.8 years (N=1) - Partial postlesional epilepsia with spastic hemiplegia after postnatal meningitis (N=1) |
| Chemotherapy + Surgery | - Delayed language development with school problems in one child of a pair of twins, at the age of 6 years (N=1) |
| Chemotherapy + Radiotherapy | / |
| Chemotherapy + Surgery + Radiotherapy | / |
| Surgery | / |
| Radiotherapy | / |
| Surgery + Radiotherapy | - Spastic diplegia with delayed psychomotor and cognitive development, without other neurologic or intracranial abnormalities, remarked progressively throughout development (N=1) |
| No treatment | - Delayed language development at the age of 6 years (N=1) - Symptoms of developmental coordination disorder (DCD), determined by a psychomotor physiotherapist at the age of 5.8 years, with watchful waiting policy for a period of therapy, in the child with congenital hip subluxation (N=1) |

*Pediatric examination was performed in 103/132 study children. Pediatric examination was not performed in two centers (2nd Medical Faculty Charles University Prague (N=7), San Gerardo Hospital Milan (N=6)). Missing data of other children (N=16) is due to preference of parents to take part only in neuropsychological assessment at home due to practical difficulties to come to the hospital.

# 2. References

1. van der Giessen, P.H., *Peridose, a software program to calculate the dose outside the primary beam in radiation therapy.* Radiother Oncol, 2001. **58**(2): p. 209-13.

2. Geijteman, E.C., et al., *A child with severe hearing loss associated with maternal cisplatin treatment during pregnancy.* Obstet Gynecol, 2014. **124**(2 Pt 2 Suppl 1): p. 454-6.

3. Amant, F., et al., *Long-term cognitive and cardiac outcomes after prenatal exposure to chemotherapy in children aged 18 months or older: an observational study.* Lancet Oncol, 2012. **13**(3): p. 256-64.
